# Histone H3 and TORC1 prevent organelle dysfunction and cell death by promoting nuclear retention of HMGB proteins

**DOI:** 10.1186/s13072-016-0083-3

**Published:** 2016-08-17

**Authors:** Hongfeng Chen, Jason J. Workman, Brian D. Strahl, R. Nicholas Laribee

**Affiliations:** 1 Department of Pathology and Laboratory Medicine, UT Center for Cancer Research, University of Tennessee Health Science Center, Memphis, TN USA; 2 Department of Biochemistry and Biophysics, University of North Carolina at Chapel Hill, Chapel Hill, NC USA

**Keywords:** Chromatin, Histone, High mobility group B, Target of rapamycin, Apoptosis, Necrosis

## Abstract

**Background:**

How cells respond and adapt to environmental changes, such as nutrient flux, remains poorly understood. Evolutionarily conserved nutrient signaling cascades can regulate chromatin to contribute to genome regulation and cell adaptation, yet how they do so is only now beginning to be elucidated. In this study, we provide evidence in yeast that the conserved nutrient regulated target of rapamycin complex 1 (TORC1) pathway, and the histone H3N-terminus at lysine 37 (H3K37), function collaboratively to restrict specific chromatin-binding high mobility group box (HMGB) proteins to the nucleus to maintain cellular homeostasis and viability.

**Results:**

Reducing TORC1 activity in an H3K37 mutant causes cytoplasmic localization of the HMGB Nhp6a, organelle dysfunction, and both non-traditional apoptosis and necrosis. Surprisingly, under nutrient-rich conditions the H3K37 mutation increases basal TORC1 signaling. This effect is prevented by individual deletion of the genes encoding HMGBs whose cytoplasmic localization increases when TORC1 activity is repressed. This increased TORC1 signaling also can be replicated in cells by overexpressing the same HMGBs, thus demonstrating a direct and unexpected role for HMGBs in modulating TORC1 activity. The physiological consequence of impaired HMGB nuclear localization is an increased dependence on TORC1 signaling to maintain viability, an effect that ultimately reduces the chronological longevity of H3K37 mutant cells under limiting nutrient conditions.

**Conclusions:**

TORC1 and histone H3 collaborate to retain HMGBs within the nucleus to maintain cell homeostasis and promote longevity. As TORC1, HMGBs, and H3 are evolutionarily conserved, our study suggests that functional interactions between the TORC1 pathway and histone H3 in metazoans may play a similar role in the maintenance of homeostasis and aging regulation.

**Electronic supplementary material:**

The online version of this article (doi:10.1186/s13072-016-0083-3) contains supplementary material, which is available to authorized users.

## Background

In response to changing environmental conditions, such as nutrient fluxes or the presence of stress, eukaryotic cells adapt by modulating chromatin structure and their gene expression programs [1]. Chromatin alterations are largely controlled by DNA methylation, histone post-translational modifications, ATP-dependent nucleosome remodeling, histone chaperones, and histone variants [2, 3]. Oftentimes, the changes made to chromatin and the subsequent regulation of key transcriptional programs is the endpoint for such environmentally responsive chromatin pathways. In some cases, however, chromatin changes function not as an endpoint but instead they propagate this information to regulate additional nuclear and/or cytoplasmic processes [1]. Thus, signaling to and from chromatin impacts a wide range of biological activities. While the mechanisms controlling chromatin structure continue to be elucidated, how environmental information is transferred to the chromatin and transcription regulatory apparatus, and its impact on chromatin’s signaling functions, remains poorly understood.

The target of rapamycin (TOR) pathway is a highly conserved signaling cascade essential for cell growth, proliferation, and suppression of stress responses [4, 5]. TOR consists of two distinct subpathways composed of TOR complex 1 (TORC1) and TOR complex 2 (TORC2). Environmental nutrients, growth factors/mitogens, and energy specifically activate TORC1, which then stimulates numerous downstream transcriptional and translational processes regulating anabolism [5]. Simultaneously, TORC1 suppresses catabolic stress responses such as autophagy [4, 5]. Budding yeast TORC1 consists of either the Tor1 or Tor2 kinase and the Kog1, Lst8, and Tco89 subunits. While TORC1 activity is essential, yeast lacking the Tor1 or Tco89 subunits is viable. However, these mutants exhibit hypersensitivity to agents that suppress TORC1, including the specific inhibitor rapamycin, nutrient starvation, and other environmental stresses [4].

Yeast TORC1 is activated predominantly by nitrogen, in particular amino acids, which is registered by the EGO complex. EGO consists of the Ego1-3 subunits, as well as the Rag GTPases Gtr1 and Gtr2. EGO resides in the vacuole membrane where it senses luminal amino acid accumulation and then activates vacuole-localized TORC1 in response [6]. The V-ATPase complex, which is the resident proton pump in the vacuole membrane, also interacts with EGO to stimulate TORC1 as well [7]. Active TORC1 then either phosphorylates downstream effectors to mediate its biological functions, or it activates some processes directly, including transcription by RNA polymerase I and III (Pol I and Pol III) [8, 9]. TORC1 downstream effector pathways include direct phosphorylation of the Sch9 kinase to promote ribosomal biogenesis, as well as activation of the Ypk3 kinase to phosphorylate ribosomal protein S6 [10, 11]. TORC1 also phosphorylates the regulatory factor Tap42 which binds to PP2A- and PP2A-like phosphatases. Tap42 sequesters these enzymes onto the vacuole surface and restricts their access to substrates, many of which regulate nutrient stress responses [4, 12, 13]. Therefore, TORC1 relays upstream environmental information to the downstream biochemical machinery controlling cell growth and proliferation.

Emerging studies also implicate TORC1 in chromatin regulation, suggesting TORC1 may mediate environment–epigenome interactions. For example, the yeast Esa1 histone acetyltransferase is recruited in a TORC1-regulated fashion to ribosomal protein (RP) genes to promote histone acetylation and gene transcription [14]. TORC1 also regulates histone H3 lysine 56 acetylation (H3K56ac), and this process is important for TORC1-dependent transcription of the ribosomal DNA (rDNA) loci by Pol I and ribosomal RNA co-transcriptional processing [15]. Further studies of yeast rDNA regulation have determined that rDNA copy number increases in a TORC1-dependent manner which is a process that is actively opposed by the sirtuins Sir2, Hst3, and Hst4 [16]. Thus, the eukaryotic genome adaptively alters gene copy number in response to environmental stimuli through a mechanism involving TORC1-dependent epigenetic regulation.

Recently, we sought to identify histone H3 or H4 residues that exhibited genetic interactions with TORC1 and as such might function in TORC1-regulated epigenetic mechanisms. Using a rapamycin-based chemical genomics screen, we determined that a mutation of H3 lysine 37 (H3K37) was synthetically lethal when combined with decreased TORC1 signaling [17]. Because H3K37 regulates high mobility group box (HMGB) association to chromatin, we examined the fate of HMGB proteins in the H3K37 mutant. Our initial studies found that an H3K37 mutant disrupted chromatin binding of the yeast HMGB protein Nhp10, causing a significant fraction of Nhp10 to localize to the cytosol when TORC1 signaling was reduced. Increased Nhp10 cytosolic accumulation correlated with massive cell death in the H3K37 mutant when TORC1 was inhibited [17]. However, whether decreased chromatin binding by Nhp10 or other HMGBs was the proximal cause of cell death under these conditions was not defined.

Besides histones, HMG proteins are the next most abundant protein component of chromatin [18]. The HMGB family is evolutionarily conserved from yeast to humans, and they function in all genome-regulatory processes by binding to minor groove DNA to create altered DNA structures [18]. While some HMGB domain-containing transcription factors bind DNA in a sequence-specific fashion, non-transcription factor HMGBs bind in a sequence-independent, but chromatin context-dependent manner. HMGBs also have additional regulatory roles independent of DNA binding. For instance, necrotic mammalian cells release the prototypical HMGB factor, HMGB1, into the extracellular milieu. This extracellular HMGB1 stimulates inflammatory processes by binding to Toll-like and RAGE receptors on innate immune cells [18]. Additionally, cytosolic HMGB1 has important roles in regulating mitochondrial function and cellular metabolism [19, 20]. Intriguingly, in vitro binding assays using HMGB1 and nucleosomal DNA demonstrate that HMGB1 selectively makes contacts with the H3 tail at multiple positions, including histone H3 lysine 36 (H3K36) and H3K37 [21, 22]. Although performed in vitro, these studies reinforce the possibility that the functional genetic interactions identified between TORC1 and H3K37 involve HMGB chromatin binding.

The mechanisms underlying the connections between HMGB chromatin binding and TORC1 have remained unclear. In this study, we provide further evidence that TORC1 and H3K37 function synergistically to retain specific HMGBs in the nucleus. Impairment of both TORC1 signaling and H3K37 causes these HMGBs to accumulate in the cytoplasm. Once cytoplasmic, these HMGBs induce both an atypical mitochondrial-dependent apoptosis and necrosis caused by vacuole dysregulation and impaired pH homeostasis. Surprisingly, we find that either reduced HMGB chromatin binding or HMGB dysregulation increases TORC1 signaling which severely curtails the chronological aging process. These results demonstrate that TORC1 signaling and H3K37 act to maintain cellular homeostasis and promote longevity by restricting HMGB localization to the nucleus.

## Results

### Histone H3K37 disruption differentially affects the nuclear localization of specific HMGBs

**Fig. 1:**
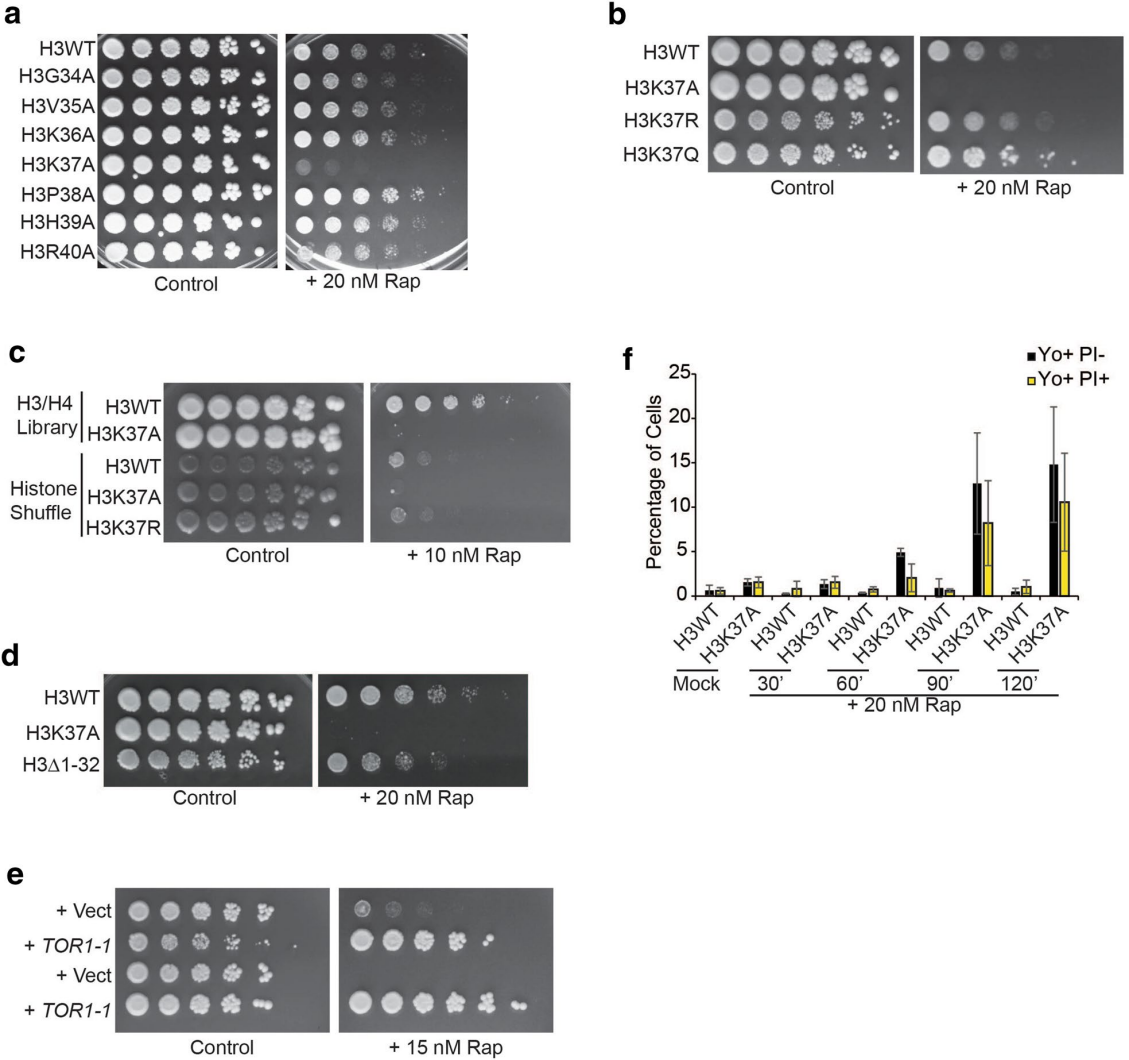
H3K37 is required for cell death suppression in TORC1-inhibited cells independent of histone modification status. **a** Spotting assay with H3WT and the indicated histone H3 mutants on control plates or plates containing 20 nM rapamycin. **b** As in **a** except a mutant that restores electrostatic charge (H3K37R) or mimics constitutive acetylation (H3K37Q) was analyzed. **c** H3WT and H3K37A mutants from **a** were spotted in parallel with a histone shuffle strain containing as the sole source of histone H3 either H3WT, H3K37A, or H3K37R. **d** As in **a**, except H3WT, H3K37A, and an H3 mutant lacking the first 32N-terminal amino acids (H3Δ1-32) were analyzed. **e** H3WT and H3K37A expressing control vector or vector expressing the rapamycin-resistant *TOR1*-*1* allele were spotted to plasmid-selective media (SC-Leu) or media containing 15 nM rapamycin. **f** H3WT and H3K37A cells were mock treated or 20 nM rapamycin treated for the indicated times and then stained with YO-PRO-1 to detect apoptosis (Yo+ PI−) and propidium iodide to detect necrosis (Yo+ PI+). Cells were then analyzed by flow cytometry

Our rapamycin-based genetic screens determined that H3K37A sensitized cells to reduced TORC1 activity [17]. This effect is solely specific for H3K37A as mutation of flanking residues, including the adjacent methylated H3K36, has no effect (Fig. 1a). H3K37A rapamycin sensitivity is independent of H3K37 post-translational modification since both H3K37R and H3K37Q restore growth on rapamycin plates, albeit H3K37Q mutants do have a modest growth advantage under these conditions (Fig. 1b). To confirm these results are not due solely to the histone H3/H4 library background, we utilized a well-characterized histone shuffle strain and shuffled into it vectors expressing H3 wild type (H3WT), H3K37A, or H3K37R as the sole source of histone H3 [23]. These cells, along with the H3WT and H3K37A derived from the library, were spotted to control or 10 nM rapamycin plates. The shuffle strain expressing H3WT is considerably more rapamycin sensitive than the H3WT from the library; however, in the shuffle background the H3K37A is still sensitive to TORC1 inhibition, while the H3K37R restores growth (Fig. 1c). These results suggest that the TORC1 phenotype caused by H3K37A is not due solely to loss of electrostatic charge or post-translational modification (since both H3K37R and H3K37Q rescue), but it is instead due to the impairment of a protein–protein contact at H3K37 due to the incorporation of the small hydrophobic alanine at this position. Because both arginine and glutamine have the potential to form electrostatic or polar interactions, their incorporation at H3K37 likely restores this disrupted function.

The histone H3N-terminus has several sites of post-translational modifications that contribute to a diverse array of chromatin functions, including gene transcription [24]. To address the interaction of the H3N-terminus with the TORC1 pathway, we compared the rapamycin sensitivity of H3WT, H3K37A, and an H3N-terminal truncation mutant (H3Δ1-32) lacking the majority of post-translationally modified N-terminal residues. H3Δ1-32-expressing cells exhibit increased sensitivity to TORC1 inhibition relative to H3WT; however, they still retain the ability to grow under these conditions unlike the complete growth impairment detected with H3K37A (Fig. 1d). The growth inhibition caused by rapamycin in H3K37A is due solely to TORC1 suppression as cells expressing a rapamycin-resistant *TOR1*-*1* vector grow comparable to *TOR1*-*1*-expressing H3WT (Fig. 1e) [25]. Therefore, while post-translationally modifiable positions on the H3N-terminus likely contribute to mediating some sensitivity to TORC1 inhibition, H3K37 provides an absolutely essential, highly specific function in this regard.

We previously demonstrated that extended TORC1 inhibition in H3K37A caused cell death by necrosis [17]. To gauge how quickly cell death occurs in H3K37A, and to determine whether it is solely necrotic or whether it may encompass both necrosis and apoptosis at early stages, we mock- or 20 nM rapamycin-treated H3WT and H3K37 for increasing lengths of time. Cells were then stained with YO-PRO-1 (which stains early apoptotic cells) and propidium iodide (PI, which stains necrotic cells) and analyzed by flow cytometry [26]. No cell death occurred in mock- or rapamycin-treated H3WT or mock-treated H3K37A over the course of the experiment (Fig. 1f). After TORC1 inhibition in H3K37A, negligible apoptosis (Yo+ PI−) and necrosis (Yo+ PI+) occurred at 60 min, while both were detected by 90 min (Fig. 1f). These results indicate that decreased TORC1 signaling in H3K37A causes cytotoxicity through both apoptosis and necrosis and that this does not begin significantly until after 60-min post-TORC1 inhibition.

**Fig. 2:**
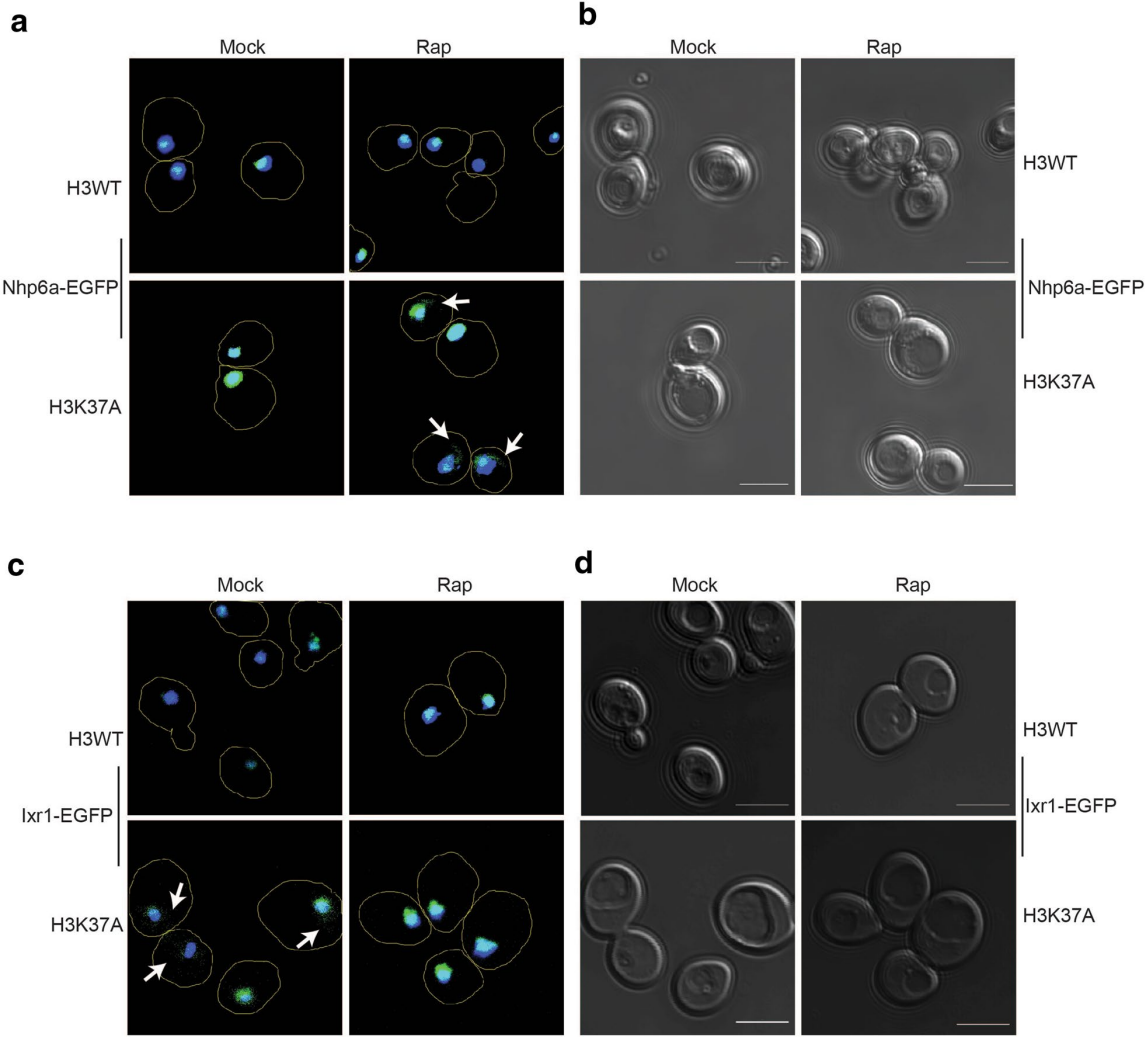
Histone H3 and TORC1 differentially regulate Nhp6a and Ixr1 cellular localization. Confocal microscopy and brightfield images of H3WT and H3K37A expressing either Nhp6a-EGFP (**a**, **b**) or Ixr1-EGFP (**c**, **d**). Cells were mock or 20 nM rapamycin treated for 1 h. The outline of individual cells is demarcated by the *line trace*. The nucleus is indicated by Hoechst (*blue*) staining. *Scale bar* indicates 5 μm for all live cell confocal images

H3K37A impairs Nhp10 chromatin binding, while TORC1 inhibition exacerbates this effect to cause Nhp10 cytoplasmic accumulation [17]. Since HMGB deregulation is cytotoxic [17], we speculated that TORC1 inhibition results in H3K37A cytotoxicity by causing HMGB cytoplasmic accumulation. To test this, we generated a series of reporter strains expressing genomically integrated EGFP reporter tags at the loci encoding the HMGBs *ABF2*, *HMO1*, *IXR1*, and *NHP6A* in H3WT and H3K37A cells. Live cell confocal microscopy of mock treated or cells treated for 1 h with 20 nM rapamycin revealed highly specific effects on HMGB cellular localization. Nhp6a was exclusively localized to the nucleus in H3WT independent of TORC1, while it was mostly nuclear in H3K37A mock-treated cells. However, in H3K37A TORC1 inhibition caused a fraction of Nhp6a to become cytosolic (Fig. 2a, b). These data were in stark contrast to those found for Ixr1. In H3WT cells, Ixr1 remained nuclear in both mock- and rapamycin-treated cells. However, in mock-treated H3K37A, Ixr1 accumulated in the cytoplasm which was reversed when TORC1 signaling was diminished (Fig. 2c, d). The HMGB Abf2 is used to demarcate mitochondria as it localizes exclusively to this organelle [27]. As expected, Abf2 localization remained in the cytoplasm in either H3WT or H3K37A irrespective of TORC1 activity, thus demonstrating the nuclear-specific effects of the histone mutation (Additional File 1: Figure S1a). Interestingly, the mitochondria in the TORC1-inhibited H3K37A cells appear to be more elongated relative to the rapamycin-treated H3WT cells, suggesting the possibility that increased mitochondrial stress may be occurring in these cells (Additional File 1: Figure S1a). Such an interpretation would be consistent with the increase in apoptotic cell death detected in TORC1-inhibited H3K37A cells (Fig. 1f) since apoptosis is a mitochondrial-dependent process [28]. Additionally, and consistent with our previous study, the nuclear localization of the TORC1 transcriptional effector HMGB, Hmo1, was unaffected under both active and reduced TORC1 signaling conditions in both H3WT and H3K37A (Additional File 1: Figure S1b) [17]. Therefore, H3K37A impairs the nuclear localization of select HMGB factors under both normal and reduced TORC1 signaling conditions.

**Fig. 3:**
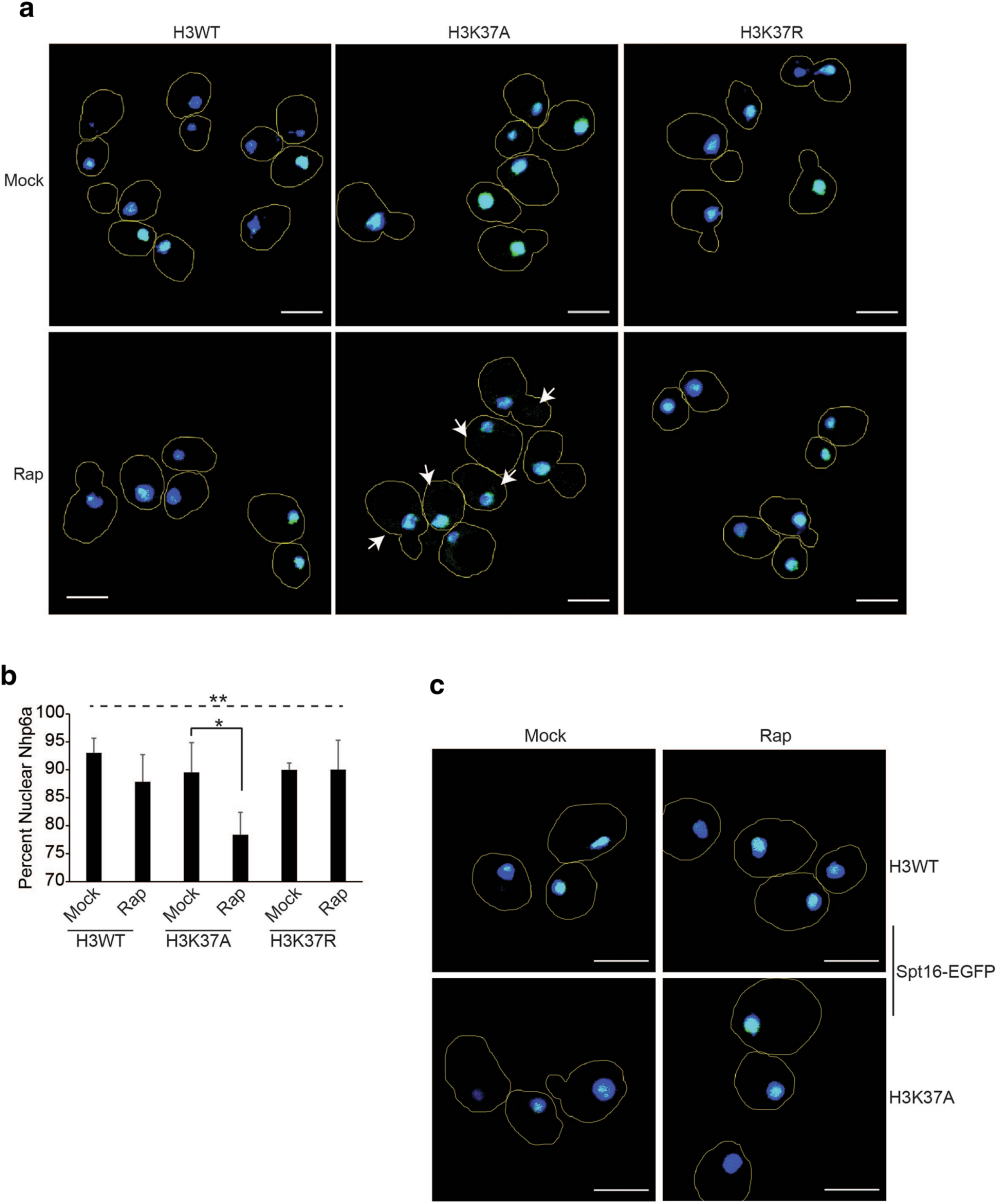
TORC1 and histone H3 regulate Nhp6a nuclear localization independently of the FACT histone chaperone complex. **a** Mock or 1-h 20 nM rapamycin-treated H3WT, H3K37A, and H3K37R cells expressing Nhp6a-EGFP were analyzed by confocal microscopy. Cell outlines are indicated by the *line trace*. **b** Quantification of Nhp6a nuclear localization from three independent experiments with the average and standard deviation (SD) plotted. One-way ANOVA was performed across all categories which is indicated by the *dashed line*, while the *solid black line* indicates the pairwise comparison analyzed by Student’s *t* test. *P < 0.05; **P < 0.01. **c**. As in **a**, except cells expressing Spt16-EGFP were analyzed. Brightfield images for **a** and **c** are in Additional File 1: Figure S3

Because TORC1 inhibition in H3K37A increased Nhp6a-EGFP cytoplasmic localization, which correlated with induction of cell death, we analyzed this HMGB further. Nhp6a-EGFP strains, along with cells expressing Nhp6a-EGFP in an H3K37R background, were cultured to log phase and either mock treated or treated with 20 nM rapamycin for 1 h, before analysis by confocal microscopy. As expected, Nhp6a localized exclusively to the nucleus in H3WT regardless of TORC1 activity, while rapamycin treatment reduced (by ~15 %) the nuclear Nhp6a pool in H3K37A (Fig. 3a, b). The H3K37R, which restores growth under impaired TORC1 signaling conditions (Fig. 1b, c), completely restored Nhp6a nuclear localization (Fig. 3a, b). To unequivocally confirm these effects on Nhp6a localization were due solely to TORC1 inhibition, we transformed H3WT and H3K37A Nhp6a-EGFP-expressing cells with control or rapamycin-resistant *TOR1*-*1* expression vector. These cells were cultured in selective, nutrient-defined media buffered to pH 6.5 and then mock or 20 nM rapamycin treated for 2 h before confocal microscopy analysis. Consistent with our previous results, Nhp6a nuclear localization was unaffected in H3WT expressing control or *TOR1*-*1* expression vector under either condition (Additional File 1: Figure S2a-b). Rapamycin-treated H3K37A cells with control vector exhibited increased Nhp6a movement to the nuclear periphery and into the cytoplasm, while the expression of the rapamycin-resistant *TOR1*-*1* allele completely restored Nhp6a nuclear localization (Additional File 1: Figure S2a-b). This effect correlates with the restoration of cell viability as well (Fig. 1e). Nhp6a and its paralog Nhp6b are redundant components of the FACT histone chaperone [29, 30]. To determine whether rapamycin-induced Nhp6a cytoplasmic localization involved FACT, we integrated an EGFP tag at the *SPT16* genomic locus (a core FACT subunit) in H3WT and H3K37A and repeated these experiments. Spt16 remained nuclear in both H3WT and H3K37A, irrespective of TORC1 activity (Fig. 3c). Therefore, H3K37A causes a subpopulation of Nhp6a not affiliated with FACT to localize to the cytoplasm when TORC1 activity is limiting, suggesting Nhp6a cytoplasmic accumulation may be connected to cell death induction.

### TORC1 inhibition in H3K37A induces cell death through both mitochondrial and vacuole dysfunction

**Fig. 4:**
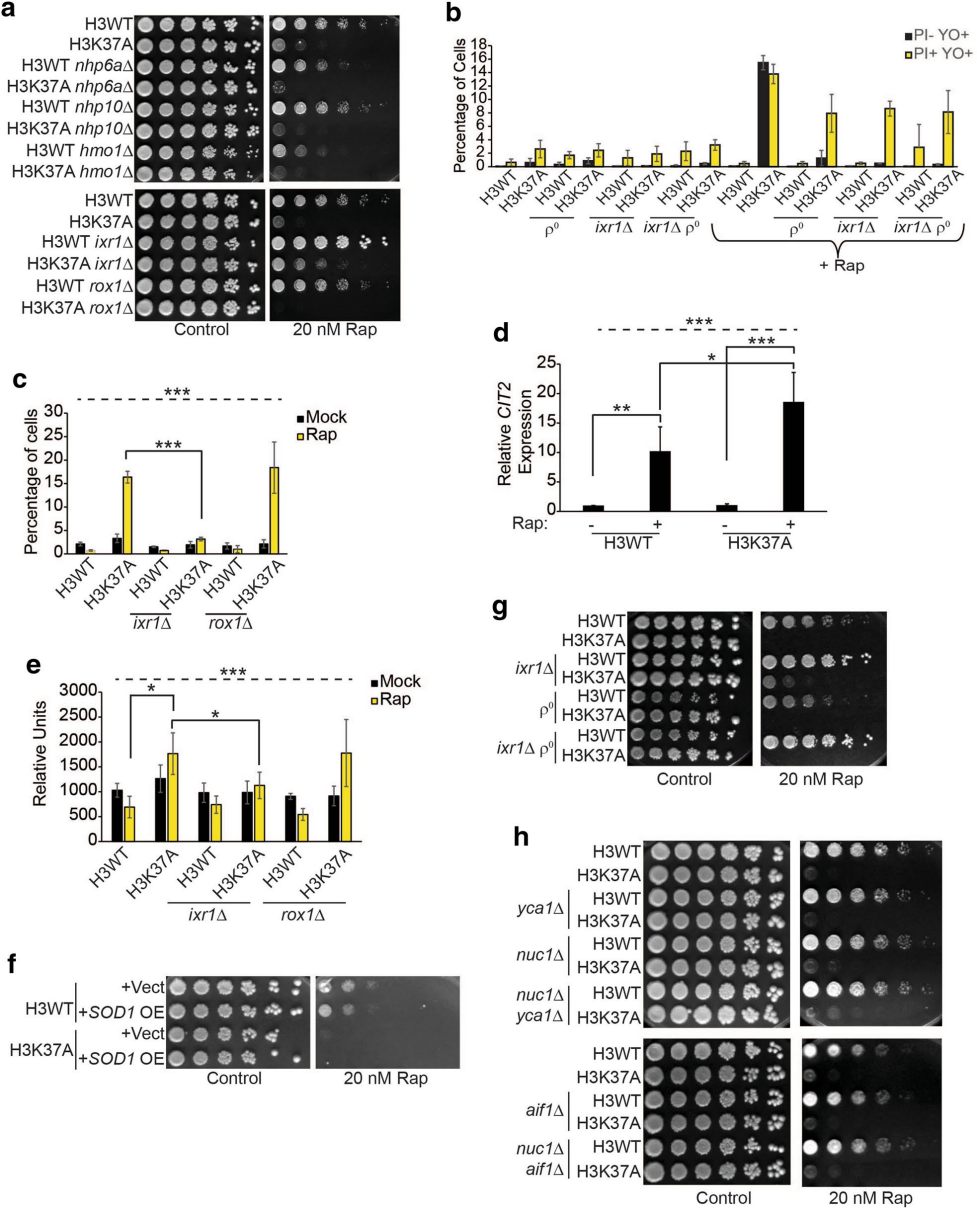
Histone H3 and TORC1 synergistically suppress both apoptosis and necrosis. **a** Spotting assay with H3WT, H3K37A, and the indicated HMG gene deletions. **b** Flow cytometry analysis of the indicated strains cultured to log phase and then mock treated or treated with 20 nM rapamycin for 5.5 h before staining with YO-PRO-1 and PI. Data are the average and SD of three independent experiments. **c** As in **b** except staining was performed only with YO-PRO-1 to solely detect apoptotic cells. **d** cDNA samples from H3WT and H3K37A mock or 20 nM rapamycin treated for 1 h were analyzed for *CIT2* expression. Data are the average and SD of five independent experiments. **e** As in **b** except cells were stained with DHE. The average and SD of three independent experiments are presented. **f** Spotting assay on selective media (SC-Leu) with H3WT and H3K37A carrying either a control vector or an *SOD1* high copy expression vector (OE). **g** Spotting assay with H3WT, H3K37A, and their derivatives lacking Ixr1 (*ixr1∆*), functional mitochondria (*ρ*°), or both. **h** As in **a** except genes encoding the indicated apoptotic effectors were deleted either individually or in combination. For all statistical analyses, one-way ANOVA was performed across all categories which is indicated by the *dashed line*, while the *solid black lines* indicate the specific pairwise comparisons which were analyzed by Student’s *t* test. *P < 0.05; **P < 0.01; ***P < 005

If cytoplasmic localization of any single HMGB induces cell death in H3K37A TORC1-inhibited cells, then loss of this HMGB may rescue growth of these cells. To test this possibility, we deleted five of the seven HMGB encoding genes in both H3WT and H3K37A. We were unable to obtain an *ABF2* gene deletion for unknown reasons, and the *NHP6B* gene, which encodes the Nhp6a paralog, overlaps with an uncharacterized gene, so it was not tested. Of the five HMGB deletions examined, only *ixr1∆* weakly rescued H3K37A growth on rapamycin plates (Fig. 4a). Intriguingly, *ixr1∆* also resulted in more robust growth of H3WT when TORC1 was inhibited (Fig. 4a), suggesting its loss provided a generalized growth advantage. These results were surprising since Ixr1 completely localizes to the nucleus in H3K37A TORC1-inhibited cells (Fig. 2b). To further characterize the mechanism involved, we cultured H3WT, H3K37A, and their *ixr1∆* derivatives to log phase, mock treated or treated with 20 nM rapamycin for 5.5 h, and then stained cells to quantify the population of both apoptotic and necrotic cells. We also engineered mitochondria-deficient (*ρ*°) derivatives to determine whether the cell death required mitochondria since this organelle regulates apoptosis [28]. TORC1 inhibition had no effect on H3WT or its derivatives, while it induced both apoptosis and necrosis in H3K37A (Fig. 4b). Individual loss of either functional mitochondria or *ixr1∆* completely abolished apoptosis in TORC1-inhibited H3K37A cells, while having negligible effects on necrosis (Fig. 4b). This necrosis was not due to parallel roles for mitochondria and Ixr1 in cell death regulation since H3K37A *ixr1∆ ρ*° cells exhibited comparable amounts of necrosis relative to individual H3K37A *ρ*° or H3K37A *ixr1∆* cells (Fig. 4b).

Both Ixr1 and the HMGB Rox1 repress genes encoding mitochondrial components [31]. To test how specific the *ixr1∆*-dependent apoptosis suppression was, we repeated the mock and 5.5-h rapamycin treatment and compared apoptosis levels in H3WT, H3K37A, and either the *ixr1∆* or *rox1∆* in these backgrounds. TORC1 inhibition induced apoptosis in H3K37A, which was suppressed by *ixr1∆*, whereas *rox1∆* had no effect (Fig. 4c). These results suggest that apoptosis suppression in *ixr1∆* is likely not due to altered transcription of Ixr1 and Rox1 co-regulated genes. Therefore, both mitochondria and Ixr1 regulate apoptosis in TORC1-limited cells, while neither affects the concomitant necrosis that occurs.

Reduced TORC1 activity increases mitochondrial function in part because the TORC1 subunit, Lst8, functions in retrograde signaling [32]. Because H3K37A rapamycin-induced apoptosis requires mitochondria, we investigated whether TORC1 inhibition in H3K37A altered retrograde signaling. Log phase cultures of H3WT and H3K37A were either mock or 20 nM rapamycin treated for one hour before analyzing expression of the retrograde-inducible *CIT2* gene [33]. Consistent with the increased mitochondrial function that occurs when TORC1 is inhibited, *CIT2* was upregulated approximately tenfold in rapamycin-treated H3WT cells compared to control (Fig. 4d). While H3K37A had no effect on *CIT2* expression in mock-treated cells, *CIT2* was induced to significantly higher levels in rapamycin-treated H3K37A relative to the comparable H3WT (Fig. 4d). Since retrograde activation occurs as a consequence of mitochondrial dysfunction, we repeated this experiment and stained cells with dihydroethidium (DHE) to measure reactive oxygen species (ROS) as an indicator of mitochondrial stress. We analyzed the derivative *ixr1∆* and *rox1∆* mutants as well. Mock-treated H3WT and H3K37A exhibited similar ROS levels, while TORC1 inhibition in H3WT did not alter ROS; however, rapamycin treatment did induce significantly higher ROS in H3K37A (Fig. 4e). Intriguingly, *ixr1∆*, but not *rox1∆*, completely suppressed this increased ROS which also correlated with *ixr1∆*-dependent suppression of apoptosis (Fig. 4b, e). To test whether this ROS caused cell death, we transformed H3WT and H3K37A cells with control vector or a multicopy vector overexpressing superoxide dismutase *SOD1*, which detoxifies ROS [34]. Surprisingly, *SOD1* overexpression did not rescue H3K37A growth when TORC1 was inhibited (Fig. 4f). While these data demonstrate a role for both mitochondrial dysfunction and Ixr1 in regulating TORC1-inhibited H3K37A apoptosis, the results suggest ROS suppression alone is insufficient to restore cell growth.

An *ixr1∆*, or loss of functional mitochondria, abolished rapamycin-induced H3K37A apoptosis, yet the *ixr1∆ ρ*° did not substantially reduce necrosis (Fig. 4b). To determine whether the double mutant enhanced the weak growth rescue that the *ixr1∆* provided H3K37A cells, we spotted these cells from Fig. 4b to control or 20 nM rapamycin plates. Consistent with our previous results (Fig. 4a), H3WT *ixr1∆* cells grew more robustly on rapamycin-containing media (evident by larger colony size), whereas H3WT *ρ*° cells grew more poorly (Fig. 4g). Surprisingly, while the H3K37A *ixr1∆* weakly restored growth, neither the H3K37A *ρ*° nor the H3K37A *ixr1∆ ρ*° mutant was capable of growth even though both suppressed apoptosis (Fig. 4b, c, g). These data suggest that mitochondria, while responsible for the TORC1-inhibited H3K37A apoptosis, also provide a required positive function necessary for cell growth under these conditions. To further characterize the apoptosis mechanisms involved, we deleted the genes encoding mitochondrial-regulated apoptosis effectors, including metacaspase (*YCA1*), endonuclease G (*NUC1*), and apoptosis-inducing factor (*AIF1*), either individually or in combination [28]. Intriguingly, none of these mutants rescued H3K37A under TORC1-suppressive conditions (Fig. 4h). These data demonstrate that TORC1 inhibition-induced H3K37A apoptosis requires Ixr1 and mitochondria, but this apoptosis occurs independently of traditional apoptotic effectors and is genetically separable from the concurrent necrosis that occurs.

**Fig. 5:**
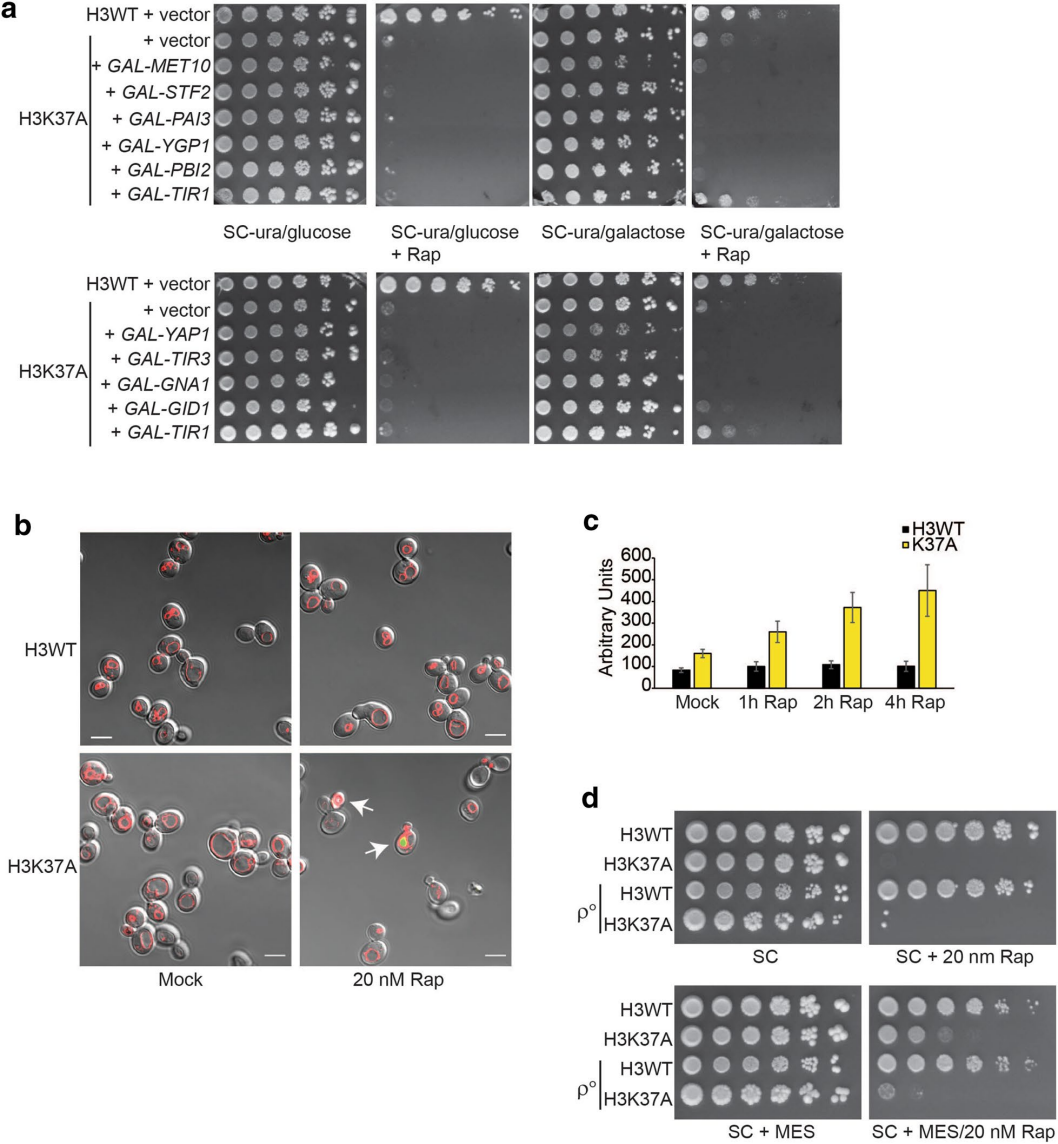
TORC1 and histone H3 prevent cell death through vacuole dysfunction and altered pH homeostasis. **a** H3WT and H3K37A transformed with control vector or the indicated galactose-inducible expression vectors were spotted to the indicated plates. **b** H3WT and H3K37A were cultured to log phase and then mock or 20 nM rapamycin treated for 30′ before staining with FM-464 (*red*, stains vacuole membrane) and 5(6)-CFDA (*green* upon acidification). *Arrows* indicate cells where significant fluorescence is detectable. **c** Experiment was performed as in **b,** and cells were sampled, stained, and analyzed by flow cytometry at the indicated times post-rapamycin treatment. Data are the average and SD of 3 independent experiments. **d** H3WT, H3K37A, and *ρ*° derivatives in these backgrounds were spotted to control synthetic complete (SC) plates or 20 nM rapamycin SC plates that were either non-buffered or buffered to pH 6.0 with MES

Loss of Ixr1 results in the transcriptional induction of many genes involved in cell metabolism and stress responses [35]. We considered the possibility that *ixr1∆* weakly rescued TORC1-inhibited H3K37A cells indirectly due to transcriptional upregulation of normally Ixr1-repressed genes. We tested this by transforming H3WT and H3K37A cells with a control vector and, for H3K37A, individual galactose-inducible vectors expressing a subset of Ixr1-repressed genes linked to metabolism (*MET10*, *PBI2*, *GNA1*, and *GID1*) or stress responses (*STF2*, *PAI3*, *TIR1*, and *TIR3*) [35]. We also included a galactose-inducible *YAP1* expression vector, since Yap1 induces stress response genes [36]. Of the genes examined, only *TIR1* overexpression weakly rescued H3K37A growth on rapamycin plates (Fig. 5a). *TIR1* encodes a cell wall mannoprotein induced under acid stress conditions [37], thus suggesting TORC1 suppression in H3K37A may induce an acid stress response to cause cell death. We tested this directly by culturing H3WT and H3K37A cells to mid-log phase and either mock or 20 nM rapamycin treated for 30 min. Cells were then stained with the vacuole-specific dye FM-464 and the pH indicator dye 5(6)-CFDA before performing confocal microscopy [38, 39]. Under physiological pH, 5(6)-CFDA does not significantly fluoresce, but as intracellular pH decreases it becomes fluorescent. Mock- and rapamycin-treated H3WT, as well as mock-treated H3K37A, exhibited no detectable 5(6)-CFDA fluorescence. However, within 30 min of TORC1 inhibition, significant 5(6)-CFDA fluorescence began to accumulate in H3K37A vacuoles indicating decreased intravacuolar pH (denotes by white arrows, Fig. 5b). This signal accumulated over time only in H3K37A, demonstrating that H3K37A TORC1 inhibition induces rapid vacuole dysfunction and altered pH homeostasis (Fig. 5c). To determine whether this intracellular acidification contributes to cell death, H3WT, H3K37A, and their *ρ*° derivatives were spotted to control and rapamycin plates which were either non-buffered or buffered to pH 6.0 with MES. Rapamycin alone prevented H3K37A growth; however, pH buffering weakly rescued H3K37A growth (Fig. 5d). Surprisingly, the H3K37A *ρ*° mutant grew more poorly under these conditions than did the H3K37A mutant (Fig. 5d). Our data suggest that while mitochondria may regulate apoptosis in H3K37A cells, they also likely provide a positive metabolic function required for cell growth when intracellular pH decreases. These results also demonstrate that the necrosis induced by TORC1 inhibition in H3K37A cells is caused by impaired vacuole homeostasis and pH dysregulation. The ability of *ixr1∆* to weakly rescue H3K37A is therefore most likely indirectly caused by the increased expression of acid stress response genes such as *TIR1* [35].

### Impaired HMGB nuclear localization dysregulates TORC1 signaling and limits chronological longevity

**Fig. 6:**
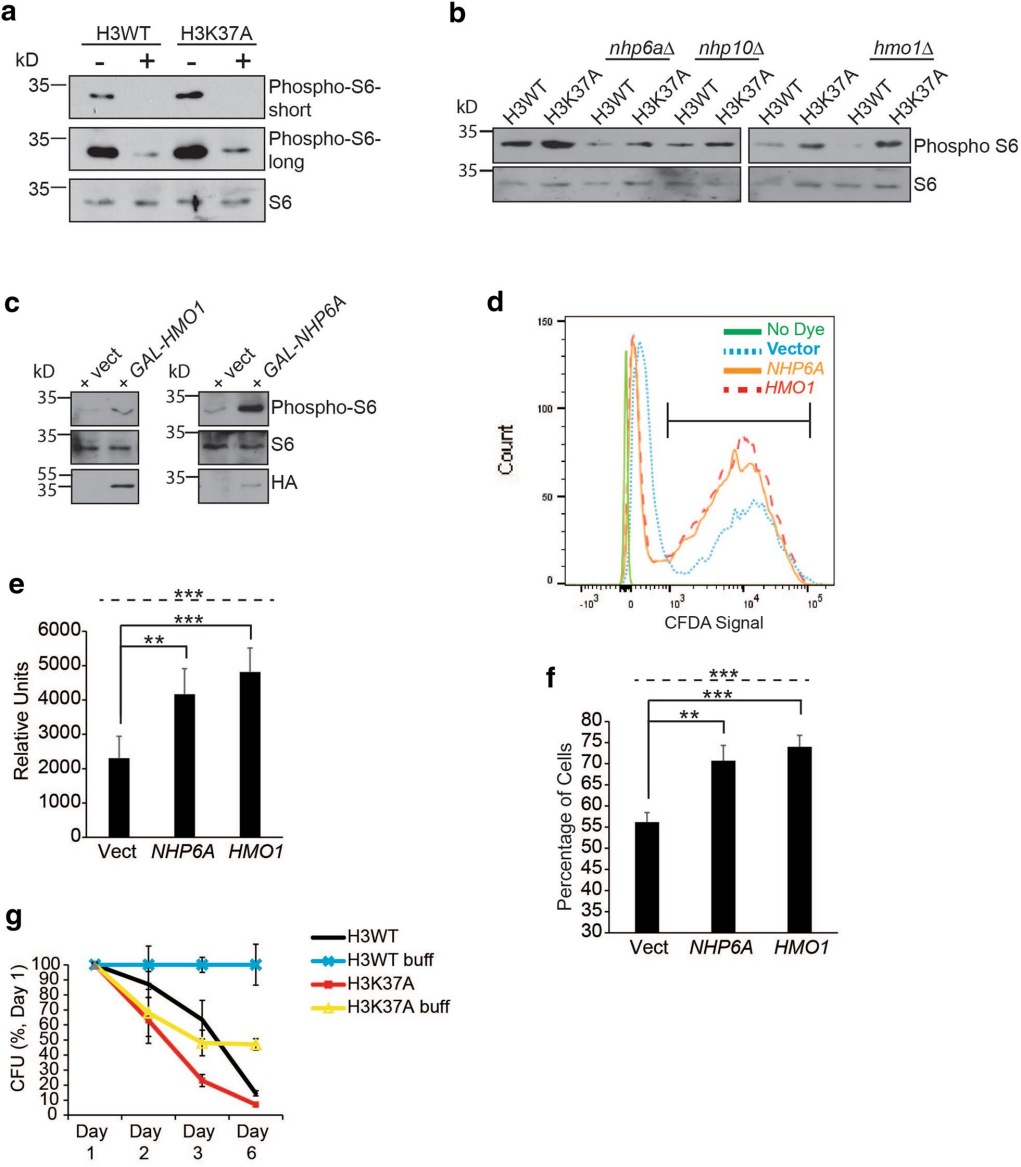
HMG chromatin binding and TORC1 function to promote cell viability and longevity. **a** IB analysis for phosphoS6 levels from H3WT and H3K37A cells cultured to log phase and then either mock or 20 nM rapamycin treated for 30 min. **b** As in **a** except the indicated HMG gene deletion mutants were included. **c** H3WT cells were transformed with control vector or the indicated galactose-regulated HMG expression vectors and cultured to log phase in raffinose media before induction with 2 % galactose for 20 min. Samples were then processed for phosphoS6 IB analysis. **d–f** As in **c** except cells were cultured solely in raffinose media to log phase and then stained with 5(6)-CFDA and analyzed by flow cytometry. **e** represents the average and SD of the peak 5(6)-CFDA fluorescence of the entire gated population, while **f** is the average cell number and SD of the fraction inside the *bracket* in **d.** Data are quantification of five independent experiments. **g** Chronological aging assay of H3WT and H3K37A performed in non-buffered and buffered (buff) SC media. Each strain was cultured in triplicate, and the data represent the average and SD of each time point performed in triplicate. For all statistical analyses, one-way ANOVA was performed across all categories (indicated by the *dashed line*), while the *solid black lines* indicate specific pairwise comparisons that were analyzed by Student’s *t* test. *P < 0.05; **P < 0.01; ***P < 005

The profound sensitivity of H3K37A cells to TORC1 inhibition suggests that they are over reliant on TORC1 activity to maintain viability. To explore this concept in greater mechanistic detail, we assessed the strength of TORC1 signaling in H3WT and H3K37A cells cultured in nutrient-rich media to mid-log phase before mock treating or treating with 20 nM rapamycin for 30 min. Cell extracts were prepared, and TORC1 activity was monitored by assessing phosphorylation of ribosomal protein S6 (phosphoS6) [11]. Surprisingly, the mock-treated H3K37A mutant exhibited substantially increased TORC1 activity relative to the comparable mock-treated H3WT, while rapamycin treatment suppressed TORC1 to approximately the same extent in both H3WT and H3K37A (Fig. 6a). To determine whether the elevated TORC1 signaling in H3K37A was due to HMGB dysregulation, we analyzed TORC1 activity in H3WT and H3K37A, as well as their *nhp6a∆* or *hmo1∆* derivatives. We also analyzed *nhp10∆* since its chromatin binding and nuclear localization are impaired in H3K37A similar to that detected for Nhp6a [17]. In the H3WT background, the *nhp6a∆*, *nhp10∆*, and *hmo1∆* decreased the overall level of TORC1 signaling relative to H3WT, suggesting these HMGBs affect basal TORC1 activity in cells with wild-type chromatin (Fig. 6b). Importantly, *nhp6a∆* and *nhp10∆*, but not *hmo1∆*, significantly reduced TORC1 signaling in H3K37A to H3WT levels (Fig. 6b). This observation is consistent with the cytoplasmic accumulation of both Nhp6a (Figs. 2a, 3a, b) and Nhp10 [17], but not Hmo1 (Additional File 1: Figure S1b) in TORC1-inhibited H3K37A cells. To test whether HMGB dysregulation directly increases TORC1 activity, we transformed H3WT cells with control or galactose-inducible HA-tagged *NHP6A* or *HMO1* expression vectors, cultured them to log phase in raffinose media, and induced them with galactose for 20 min before preparing cell extracts and analyzing phosphoS6. Hmo1 expression caused a minor, while Nhp6a expression caused a much greater, increase in phosphoS6 (Fig. 6c). These results demonstrate that HMGB dysregulation, due to impaired chromatin association or deregulated HMGB expression, stimulates TORC1 signaling.

We next examined whether HMGB dysregulation in H3WT mimicked the same defect in vacuolar pH homeostasis that occurred in H3K37A upon TORC1 limitation. We initially attempted a galactose induction utilizing our HMGB expression vectors coupled with 5(6)-CFDA staining and flow cytometry analysis. However, galactose treatment caused cell acidification even in vector control cells which confounded data interpretation (data not shown). To bypass this issue, we repeated the experiment in cells cultured solely to log phase in raffinose media since we have found these vectors exhibit low level, leaky HMGB expression. Log phase cells were stained with 5(6)-CFDA and analyzed by flow cytometry (Fig. 6d). Expression of either Nhp6a or Hmo1 increased overall mean fluorescence intensity of the population (Fig. 6e), as well as the total percentage of cells falling within the acidified category (Fig. 6f, cell population quantified is indicated by the horizontal bracket in Fig. 6d). These data demonstrate that HMGB dysregulation in cells with wild-type chromatin replicates the increased vacuolar acidification detected in TORC1-inhibited H3K37A cells.

Reduced TORC1 activity extends both chronological and replicative longevity in all organisms examined [40]. Because H3K37A increases TORC1 signaling, we specifically determined whether this histone mutant altered chronological longevity. Yeast chronological aging assays involve culturing cells in minimal (SC) media for 3 days to exhaust nutrients. At this point, the experiment is initiated (Day 0) and quantification of cell survival by analyzing colony-forming units (CFUs) begins [41]. Our initial attempts following this approach resulted in too few viable H3K37A cells at Day 0, so we modified the experiment and only cultured cells for 1 day before initiating the experiment. As media acidification can confuse interpretation of yeast aging studies [42], we performed the experiments in media either non-buffered or buffered to pH 6.0. Under non-buffered conditions, both H3WT and H3K37A exhibited decreased viability by Day 3, although H3K37A viability was reduced significantly more than H3WT (Fig. 6g). Importantly, performing the experiment in buffered media prevented loss of H3WT viability over the course of the experiment, thus demonstrating that decreased H3WT viability under non-buffered conditions is due solely to acid stress and is not a true longevity defect (Fig. 6g). However, even under buffered conditions H3K37A viability was substantially reduced (by ~50 %), thus demonstrating a true reduction in chronological longevity independent of acid stress (Fig. 6g). Collectively, these data demonstrate that the histone H3N-terminus anchors HMGBs in the nucleus to maintain normal TORC1 regulation and promote cell viability. Impairing this process deregulates TORC1 signaling and causes cytotoxicity under conditions that mimic nutrient limitation (rapamycin treatment) or reflect a nutrient-depleted state (chronological aging).

## Discussion

While it is clear that responses to environmental change include epigenetic mechanisms that depend on dynamic chromatin regulation, how this information is faithfully transmitted to chromatin is not well understood. In this report, we provide critical evidence that the nutrient regulated TORC1 pathway, and the histone H3N-terminal tail at H3K37, function collaboratively to retain specific chromatin-associated HMGB factors within the nucleus to maintain cell viability. These data provide further support for the concept that signaling through TORC1 is a key mechanism by which the environment communicates with chromatin to affect the epigenome. They also demonstrate an essential role for chromatin in restricting HMGBs to the nucleus that is required for maintaining viability in TORC1-suppressive environmental conditions, including nutrient stress or during chronological aging. Our results support the idea that under conditions which severely repress TORC1, HMGB chromatin dissociation and cytoplasmic localization may act as a cell death-initiating event. Conceptually, this idea is distinct from the current paradigm developed from studies in mammalian cells where release of HMGB1 from chromatin occurs after necrosis is initiated. This proposed function for chromatin also would be consistent with the growing recognition that chromatin modulation is not solely an endpoint for upstream signaling pathways. Instead, dynamic chromatin changes can integrate inputs from upstream signaling pathways and then propagate this information to control additional regulatory pathways to mediate biological effects. Therefore, TORC1 signaling and downstream control of HMGB chromatin association may function as a bidirectional signaling relay to mediate control of cell viability under nutrient-regulated conditions.

Our data further demonstrate that H3K37A selectively affects HMGB nuclear localization, thus illustrating that not all HMGBs are governed by the same chromatin-interacting mechanisms. For example, we demonstrate that Ixr1 steady-state nuclear localization is perturbed by H3K37A when TORC1 is not inhibited, yet under these same conditions, Nhp6a is only minimally affected, while Hmo1 is not affected at all. However, this situation is reversed when TORC1 is inhibited, such that Ixr1 becomes exclusively nuclear, while Nhp6a accumulates in the cytoplasm. These results, coupled with our previous observation that H3K37A decreases Nhp10 chromatin binding, suggest that in vivo, H3K37 stabilizes specific HMGB interactions with chromatin. Such a scenario is consistent with the highly selective role for the histone H3N-terminal tail in contacting HMGB1 in in vitro nucleosomal binding assays [21, 22]. This study further strengthens the concept that chromatin can dictate HMGB association in vivo, an idea which had been suggested previously from genome-wide and gene-specific HMGB analyses [17, 43]. Because HMG proteins constitute the largest fraction of non-histone chromatin-associated proteins, further defining the mechanisms governing their chromatin binding will be essential for understanding their role in genome regulation.

Additional mechanisms likely function in parallel with H3K37 to promote HMGB chromatin association and nuclear localization, including redundant interactions with the H3 tail, specific chromatin structures regulated by the local histone post-translational modification environment, or even modification of the HMGBs directly. This latter point is especially relevant since HMGB1 is extensively modified by a variety of post-translational modifications including phosphorylation, acetylation, and oxidation [44, 45]. A distinct possibility is that TORC1 regulates HMGB modifications to facilitate their chromatin binding such that combining H3K37A with decreased TORC1 signaling synergistically impairs HMGB chromatin association and nuclear retention. H3K37 disruption, while selectively impacting HMGB nuclear localization, clearly has differential effects on these HMGBs. The mechanisms driving these differences are not immediately obvious but could be related to the distinct functions of each HMGB. For example, Ixr1 has a potential role as a stress response regulator since it functions both as a transcriptional activator and as repressor of many target genes involved in this process [35]. Since TORC1 inhibition activates the environmental stress response, TORC1 repression may restore Ixr1 nuclear localization in H3K37A by overriding the inhibitory effect H3K37A has on Ixr1 chromatin binding [46]. While H3K37A minimally affects Nhp6a nuclear localization during normal growth, suppressing TORC1 causes a fraction of Nhp6a to localize to the cytoplasm. How the majority of Nhp6a remains in the nucleus under these conditions is unclear. However, Nhp6a is an abundant HMGB so a distinct possibility could be that the cytoplasmic Nhp6a pool in TORC1-inhibited H3K37A cells may derive from a normally “hyperdynamic” fraction of genome-bound, FACT-independent Nhp6a. In environmental conditions that repress TORC1, chromatin may anchor this hyperdynamic HMGB population in the nucleus to prevent its cytoplasmic localization and induction of cell death.

We believe that disrupted HMGB chromatin association, and their consequent cytoplasmic localization, likely initiates cell death in TORC1-inhibited H3K37A cells. We base this conclusion on the following observations. The H3K37R mutation both restores Nhp6a nuclear localization and completely rescues growth when TORC1 is inhibited. Furthermore, significant cell death is only detectable after Nhp6a localizes to the cytoplasm, implying that HMGB movement to the cytoplasm occurs before cell death initiation. Supporting this concept, we and others have demonstrated that increased HMGB expression causes cytotoxicity, although the underlying mechanisms were not defined [17, 47, 48]. We provide a more detailed understanding of this process by demonstrating that intravacuolar pH dysregulation caused by TORC1 inhibition in H3K37A can be replicated in cells with wild-type chromatin solely through HMGB deregulation. Furthermore, buffering pH partially restores H3K37A growth under TORC1-suppressive conditions. These results demonstrate that cytoplasmic HMGBs impair vacuole homeostasis through unknown mechanisms to cause necrosis. The apoptosis we observed in TORC1-inhibited H3K37A cells is more difficult to interpret. Apoptosis clearly depends on functional mitochondria, although deletion of traditional apoptosis effectors is incapable of restoring growth to these cells. Although this could be due to the concurrent necrosis that occurs, H3K37A *ρ*° cells fail to grow under buffered conditions when TORC1 is inhibited. Therefore, suppressing both apoptosis and pH-induced necrosis simultaneously is not sufficient to promote growth of H3K37A cells. We interpret these data to suggest that while mitochondrial dysfunction is responsible for the apoptosis in TORC1-inhibited H3K37A, mitochondria provide additional metabolic requirements necessary for cell growth when TORC1 is impaired. Such a role for mitochondria would be consistent with the upregulation of mitochondrial function when TORC1 is inhibited [49, 50].

Finally, the TORC1 dysregulation caused by H3K37A is suppressed by *nhp6a∆* or *nhp10∆*, but not by *hmo1∆*. Nhp6a and Nhp10 are the two HMGBs whose cytoplasmic localization specifically increases when TORC1 activity is reduced, suggesting their movement to the cytoplasm contributes to TORC1 deregulation. Although the majority of Nhp6a localizes to the nucleus in H3K37A before TORC1 suppression, it is possible that a small fraction exists in the cytoplasm which cannot be reliably detected. Furthermore, while we have not specifically addressed whether *ixr1∆* suppresses the deregulated TORC1 signaling in H3K37A, this HMGB likely does contribute since a significant fraction of it is cytoplasmic during normal H3K37A growth. An intriguing observation is that while Hmo1 localization is not affected by H3K37A, and *hmo1∆* fails to reduce H3K37A TORC1 dysregulation, this HMGB is a key effector of the TORC1-regulated transcriptome [51]. Therefore, not all HMGBs linked to TORC1-regulated transcription are impacted by H3K37A. The observation that multiple HMGBs are affected by H3K37A and contribute to TORC1 deregulation does provide an explanation for why no single HMGB gene deletion restores viability to TORC1-inhibited H3K37A cells. The cytoplasmic accumulation of multiple HMGBs simultaneously is likely what induces cytotoxicity upon TORC1 suppression. How cytoplasmic HMGBs dysregulate TORC1 signaling, and this causes cell death when TORC1 activity is reduced, is not yet understood. Regardless of the mechanisms involved, our data outline a role for TORC1 and the H3N-terminal tail in HMGB nuclear anchoring. This pathway is essential for cell survival when TORC1 activity is limiting, and it plays an important role in chronological aging. In metazoans, mechanisms that alter the H3N-terminus over time in post-mitotic cells may release nuclear HMGBs into the cytoplasm to limit their chronological longevity.

## Conclusions

This study reveals a crucial role for H3K37 in the maintenance of cell homeostasis and viability under conditions of reduced TORC1 signaling. Specifically, we demonstrate that H3K37 disruption differentially effects the nuclear localization of a subset of HMGB proteins. Most importantly, we show that impaired TORC1 signaling in an H3K37 mutant increases the localization of the model HMGB, Nhp6a, and that this correlates with impaired organelle homeostasis and the induction of both apoptosis and necrosis. Intriguingly, while the apoptosis requires mitochondria, it does not depend on traditional apoptotic effectors. The resulting necrosis which occurs is connected to impaired vacuole homeostasis and pH dysregulation. Unexpectedly, our results directly show that H3K37 disruption increases basal TORC1 signaling, an effect which is suppressed by deletion of the genes encoding those HMGBs whose cytoplasmic localization increases in an H3K37 mutant. This increased TORC1 signaling can be replicated in cells with normal chromatin by overexpressing these same HMGBs, thus demonstrating a direct role for HMGBs in TORC1 deregulation. The physiological consequence TORC1 deregulation is to severely reduce the chronological longevity of the H3K37 mutant, a result consistent with TORC1 as an essential aging regulator. HMG proteins are highly conserved throughout evolution, and they constitute the most abundant protein component of chromatin outside of histones. Our results suggest the evolutionarily conserved H3N-terminal tail likely anchors HMGBs in all eukaryotes as a mechanism of retaining them within the nucleus to maintain homeostasis, prevent TORC1 deregulation, and promote cellular longevity.

## Methods

### Yeast plasmids, strains, and culture conditions

Yeast strains and plasmids utilized are listed in Additional File 2: Table S1 and Table S2, respectively. Except for the histone H3 shuffle strain used in Fig. 1c, all other yeast histone mutants in this study were derived from the published histone H3/H4 library [52] which was purchased from Open Biosystems (GE Dharmacon). To generate *ura3∆* derivatives of the H3WT and H3K37A, cells were streaked to 5-FOA-containing plates and resistant clones, which had lost the ability to grow in the absence of uracil, were isolated. Yeast strain engineering, including gene deletion or epitope tagging, was performed as described [53]. For experiments in nutrient-rich media, cells were cultured in 1 % yeast extract/2 % peptone/2 % dextrose (YPD) with media components purchased from Research Products International. Experiments in minimal media were performed by culturing cells in yeast synthetic complete (SC) media (0.17 % yeast nitrogen base/0.1 % glutamic acid/2 % glucose/0.2 % dropout mix) or SC media lacking the appropriate nutrient. Yeast SC culture media reagents were purchased from US Biologicals. To isolate H3WT and H3K37A *ρ*° mutants, cells were cultured in SC media containing 25 mg/mL ethidium bromide and then individual colonies were isolated. All *ρ*° mutants were confirmed to have lost functional mitochondria by their inability to grow on a non-fermentable (glycerol) carbon source. For the confocal microscopy analysis experiments utilizing the *TOR1*-*1* expression vector, cells were cultured to mid-log phase in SC-leucine media that was buffered to pH 6.5 before treating with 20 nM rapamycin for two hours. All cells were cultured at 30 °C with shaking. For spotting assays, equal cell numbers from overnight cultures were pelleted, washed, and then fivefold serially diluted. Cells were then spotted to the appropriate plates and incubated at 30 °C for four to six days before photographing. The H3K37A and H3K37R histone plasmids were generated via standard site-directed mutagenesis with plasmid pWZ414-F12 as a template [23]. To generate the high copy vector overexpressing *SOD1*, 300 base pairs upstream of the translational start site and 100 bp downstream of the translational stop of the SOD1 genomic locus from yeast strain BY4741 were cloned as an XhoI/BamHI fragment into vector pRS426. Galactose-regulated plasmids were purchased from Open Biosystems.

### Antibodies and cell stains

The following antibodies were utilized: α-RPS6 (Abcam), α-phosphoS6 (Cell Signaling), α-HA and α-GFP (Santa Cruz), and α-G6PDH (Sigma). DNA staining of live cells was performed with Hoechst stain (Life Technologies). YO-PRO-1 and PI stains were purchased from Life Technologies.

### Live cell confocal microscopy

For the GFP localization experiments, cells were grown to log phase, pelleted, and washed twice with sterile water. Pellets were resuspended in 100 μL sterile water and stained with Hoechst 33342 (2 μg/mL) for 20–30 min prior to mounting onto polylysine-coated slides and confocal analysis. The vacuole pH experiments were conducted similarly to the GFP localization experiments, except FM4-64 (8 μM) and CFDA (5 μM) were added to cells cultured in YPD.

### Image analysis in Zen 2 blue

Zen Lite version 2.0.0 software was utilized to perform quantification of Nhp6a nuclear/cytoplasmic localization. The Spline Contour tool was used to trace borders around the cell periphery and nucleus of individual cells. Borders were closed, and the fluorescence intensity within each enclosed area was calculated, along with the mean intensity value for each channel. The nuclear area was multiplied by the mean intensity value for the green channel to give the total nuclear fluorescence intensity (TNFI).

$${\text{TNFI = nuclear}}\;{\text{area}} \left( {{\text{nm}}^{2} } \right)\;*\;{\text{nuclear}}\;{\text{mean}}\;{\text{intensity}}\;{\text{value}}$$

This was repeated using the outer cell border values which provided a measure of the total cellular fluorescence intensity (TCFI).

$${\text{TCFI}} = {\text{cellular}}\;{\text{area}} \left( {{\text{nm}}^{2} } \right)\;*\;{\text{cellular}}\;{\text{mean}}\;{\text{intensity}}\;{\text{value}}$$

The following calculation was then performed to get the percentage of total nuclear protein:

$$\% {\text{nuclear}} = \frac{\text{TNFI}}{\text{TCFI}}\;*\;100$$

Random fields of cells were chosen for quantification, with approximately 20–40 cells quantified per condition, per biological replicate (3 replicates). Only cells with clear nuclear DNA staining and detectable EGFP signal were quantified.

### Flow cytometry

For the YO-PRO-1 and PI staining, cells were grown to log phase, pelleted, and washed twice with sterile PBS. YO-PRO-1 (10 μM) and PI (50 μg/mL) were added followed by a 20- to 30-min incubation. Samples were then processed on a BD LSRII flow cytometer, and data were analyzed using FlowJo V10. DHE (30 μM) and 5(6)-carboxyfluorescein diacetate (CFDA, 100 μM) staining were performed identically to that described above.

### RT-qPCR and immunoblot analysis

Total RNA was extracted, and randomly primed cDNA was synthesized using 1 μg of DNase I-digested RNA and the ImProm II Reverse Transcription System from Promega. Gene-specific qPCR and normalization to the *SPT15* housekeeping gene were performed as previously described [54]. Whole-cell extracts and immunoblotting were prepared and performed as outlined previously [54]. To quantify immunoblot results, films were scanned and analyzed by ImageJ software.

### Chronological aging assay

Single H3WT or H3K37A colonies were picked from freshly streaked YPD plates and cultured overnight in 5 mL SC. The stationary phase cultures were then diluted into fresh SC media to an OD_600_ of 0.1 and returned to the incubator. This marked the beginning of the experiment, denoted as “day 0.” Because the H3K37A viability declined so quickly, dilution and spotting were conducted every day for 6 days. For the buffered media, the 50-mL SC media used to age the cells were adjusted to pH 6.0 using citrate phosphate buffer (64.2 mM Na_2_HPO4 and 17.9 mM citric acid).

## Additional files

10.1186/s13072-016-0083-3 **Figure S1.** TORC1 inhibition does not alter Abf2 or Hmo1 cellular localization. H3WT and H3K37A cells expressing Abf2-EGFP (A) or Hmo1-EGFP (B) were mock or 20 nM rapamycin treated for one hour before performing confocal microscopy analysis. The nucleus is indicated by Hoechst (blue) staining and cell outlines are indicated by the line trace. Scale bar indicates 5 μm.** Figure S2.** Rapamycin-resistant TOR1-1 expression restores nuclear Nhp6a localization in H3K37A. Nhp6a-EGFP expressing H3WT (A) or H3K37A (B) cells carrying control vector or the TOR1-1 expression vector were mock or 20 nM rapamycin treated for two hours before confocal microscopy analysis. The outline of individual cells is indicated by the line trace. Position of the nucleus is indicated by blue Hoechst staining. Arrows indicate Nhp6a at the nuclear periphery and cytoplasmic Nhp6a-EGFP signal. Scale bar indicates 5 μm.** Figure S3.** Brightfield images for Figure 3. A Brightfield images for Nhp6a-EGFP results presented in Figure 3A. B Brightfield images for the Sp16-EGFP data represented in Figure 3C. Scale bar indicated 5 μm.
10.1186/s13072-016-0083-3 **Table S1.** Yeast strains. **Table S2.** Yeast plasmids.

## Abbreviations

TORC1: target of rapamycin complex 1
HMGB: high mobility group box
