# Supplementary figures and images for: Histone H3 and TORC1 prevent organelle dysfunction and cell death by promoting nuclear retention of HMGB proteins
Source: Epigenetics Chromatin. 2016 Aug 17;9:34. doi: 10.1186/s13072-016-0083-3 (PMC4989345; doi:10.1186/s13072-016-0083-3)

**Figure S1.**

**A**

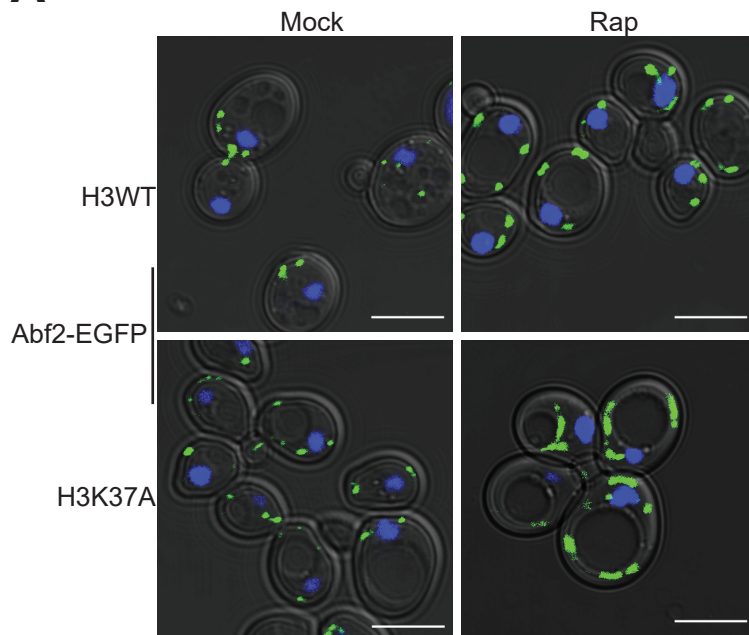

**B**

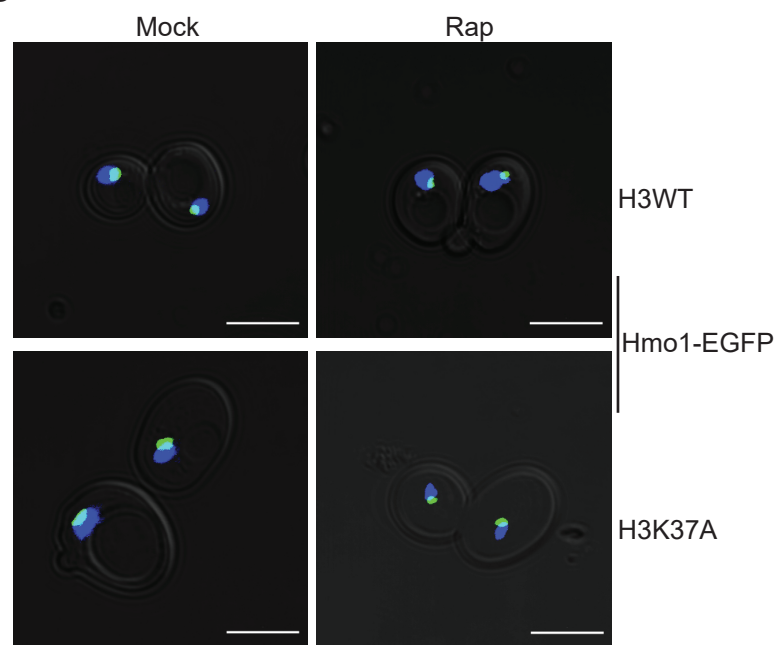

**Figure S2.**

**A**

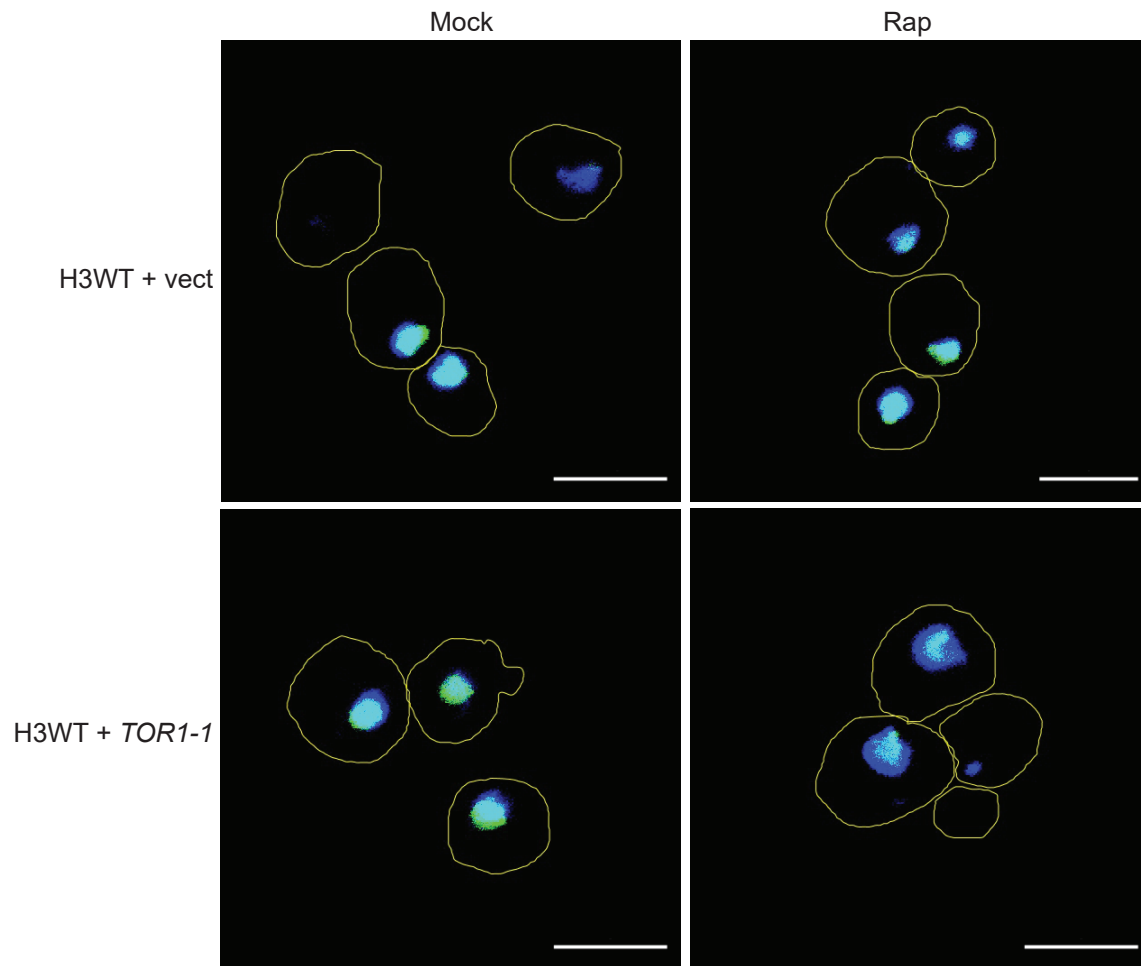

**B**

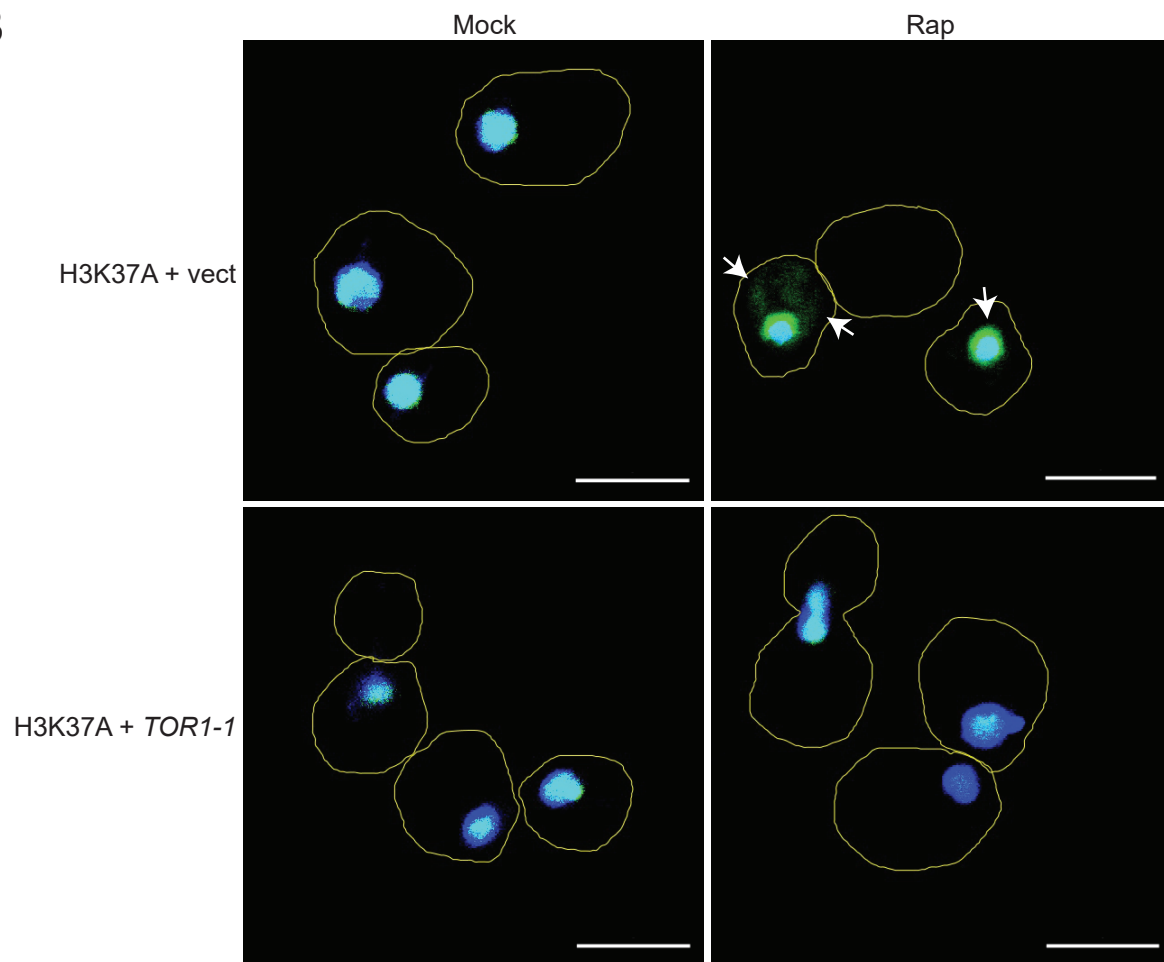

**Figure S3.**

**A**

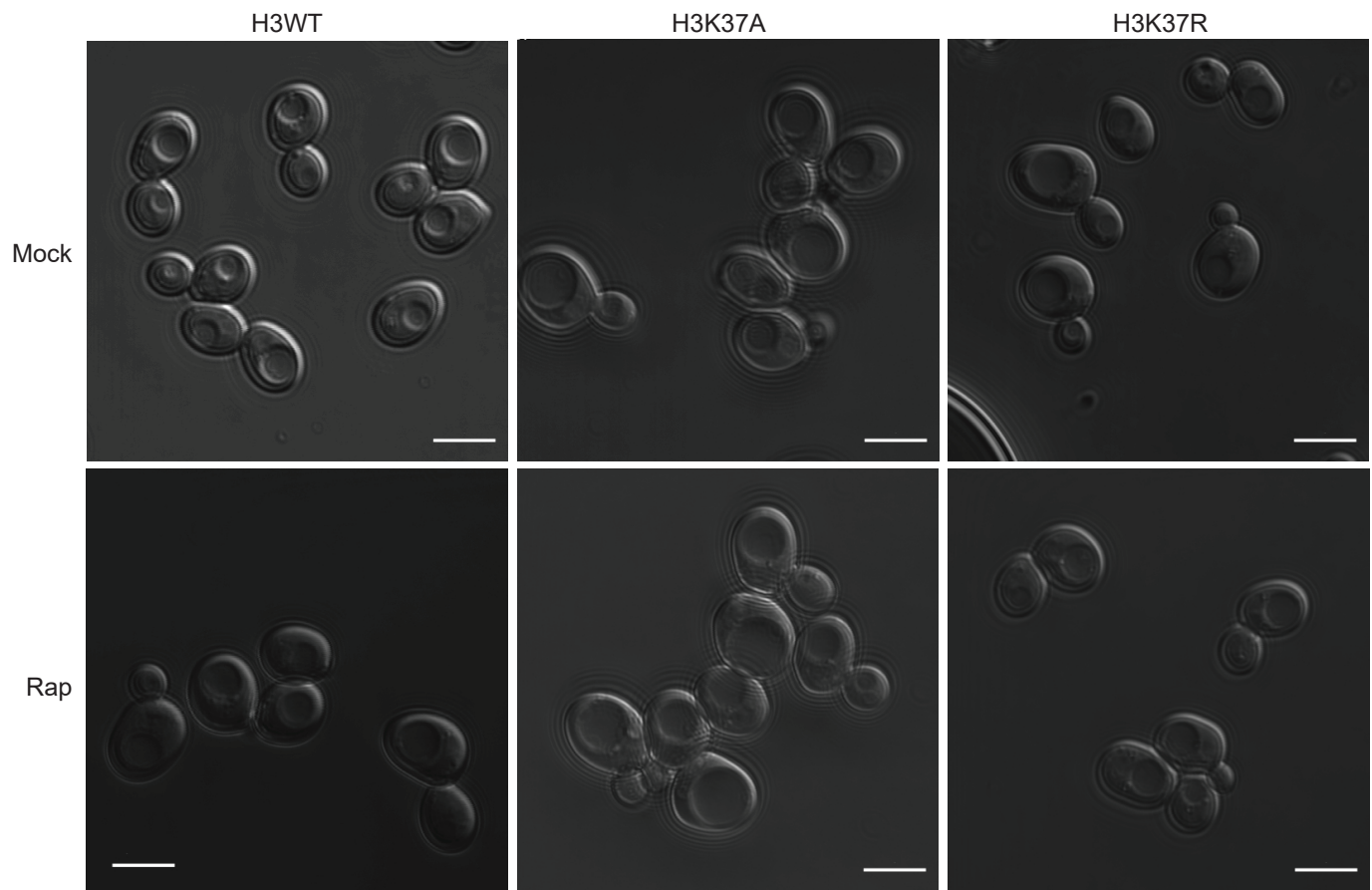

**B**

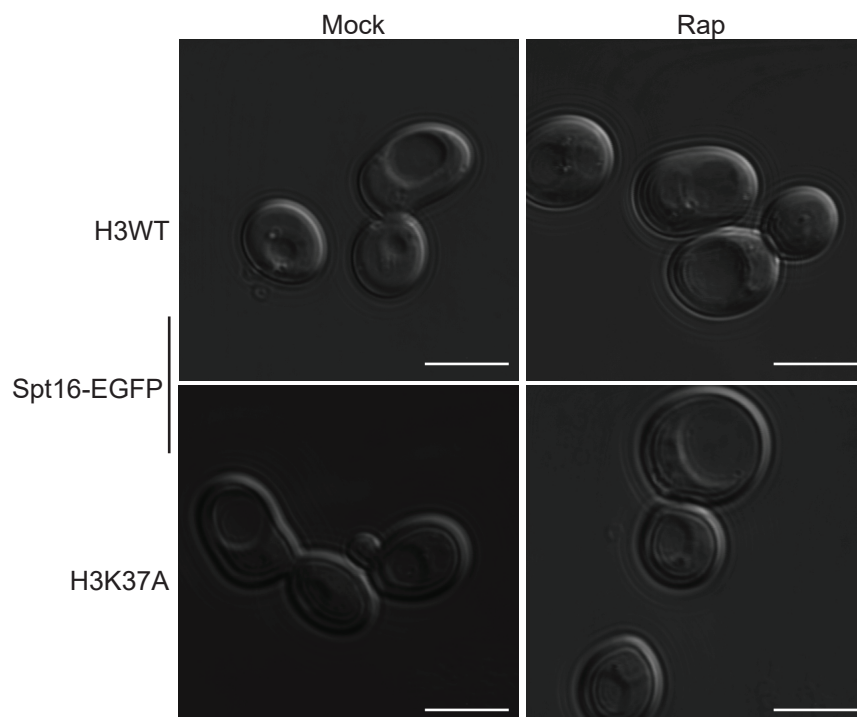

Supplement: Supplementary file 1 — 10.1186/s13072-016-0083-3 Figure S1. TORC1 inhibition does not alter Abf2 or Hmo1 cellular localization. H3WT and H3K37A cells expressing Abf2-EGFP (A) or Hmo1-EGFP (B) were mock or 20 nM rapamycin treated for one hour before performing confocal microscopy analysis. The nucleus is indicated by Hoechst (blue) staining and cell outlines are indicated by the line trace. Scale bar indicates 5 μm. Figure S2. Rapamycin-resistant TOR1-1 expression restores nuclear Nhp6a localization in H3K37A. Nhp6a-EGFP expressing H3WT (A) or H3K37A (B) cells carrying control vector or the TOR1-1 expression vector were mock or 20 nM rapamycin treated for two hours before confocal microscopy analysis. The outline of individual cells is indicated by the line trace. Position of the nucleus is indicated by blue Hoechst staining. Arrows indicate Nhp6a at the nuclear periphery and cytoplasmic Nhp6a-EGFP signal. Scale bar indicates 5 μm. Figure S3. Brightfield images for Figure 3. A Brightfield images for Nhp6a-EGFP results presented in Figure 3A. B Brightfield images for the Sp16-EGFP data represented in Figure 3C. Scale bar indicated 5 μm. [file 13072_2016_83_MOESM1_ESM.pdf]
