# Supplementary material for: Histone H3 and TORC1 prevent organelle dysfunction and cell death by promoting nuclear retention of HMGB proteins
Source: Epigenetics Chromatin. 2016 Aug 17;9:34. doi: 10.1186/s13072-016-0083-3 (PMC4989345; doi:10.1186/s13072-016-0083-3)
Supplement: Supplementary file 2 — 10.1186/s13072-016-0083-3 Table S1. Yeast strains. Table S2. Yeast plasmids. [file 13072_2016_83_MOESM2_ESM.docx]

**Supplemental Table S1. Yeast strains.**

| **Strain** | **Genotype** | **Source** |
| --- | --- | --- |
| BY4741 | MATa *his3Δ1 leu2Δ0 met15Δ0 ura3Δ0* BY4741 | OPEN Bio |
| H3WT | MATa *his3Δ200 leu2Δ0 lys2Δ0 trp1Δ63 ura3Δ0met15Δ0 can1::MFA1pr-HIS3 hht1-hhf1::NatMX4 hht2-hhf2::[HHTS-HHFS]- ura3Δ* H3WT | OPEN Bio |
| H3G34A | MATa *his3Δ200 leu2Δ0 lys2Δ0 trp1Δ63 ura3Δ0met15Δ0 can1::MFA1pr-HIS3 hht1-hhf1::NatMX4 hht2-hhf2::[HHTS-HHFS]- ura3Δ* H3G34A | OPEN Bio |
| H3V35A | MATa *his3Δ200 leu2Δ0 lys2Δ0 trp1Δ63 ura3Δ0met15Δ0 can1::MFA1pr-HIS3 hht1-hhf1::NatMX4 hht2-hhf2::[HHTS-HHFS]- ura3Δ* H3V35A | OPEN Bio |
| H3K36A | MATa *his3Δ200 leu2Δ0 lys2Δ0 trp1Δ63 ura3Δ0met15Δ0 can1::MFA1pr-HIS3 hht1-hhf1::NatMX4 hht2-hhf2::[HHTS-HHFS]- ura3Δ* H3K36A | OPEN Bio |
| H3K37A | MATa *his3Δ200 leu2Δ0 lys2Δ0 trp1Δ63 ura3Δ0met15Δ0 can1::MFA1pr-HIS3 hht1-hhf1::NatMX4 hht2-hhf2::[HHTS-HHFS]- ura3Δ* H3K37A | OPEN Bio |
| H3K37R | MATa *his3Δ200 leu2Δ0 lys2Δ0 trp1Δ63 ura3Δ0met15Δ0 can1::MFA1pr-HIS3 hht1-hhf1::NatMX4 hht2-hhf2::[HHTS-HHFS]- ura3Δ* H3K37R | OPEN Bio |
| H3K37Q | MATa *his3Δ200 leu2Δ0 lys2Δ0 trp1Δ63 ura3Δ0met15Δ0 can1::MFA1pr-HIS3 hht1-hhf1::NatMX4 hht2-hhf2::[HHTS-HHFS]- ura3Δ* H3K37Q | OPEN Bio |
| H3P38A | MATa *his3Δ200 leu2Δ0 lys2Δ0 trp1Δ63 ura3Δ0met15Δ0 can1::MFA1pr-HIS3 hht1-hhf1::NatMX4 hht2-hhf2::[HHTS-HHFS]- ura3Δ* H3P38A | OPEN Bio |
| H3H39A | MATa *his3Δ200 leu2Δ0 lys2Δ0 trp1Δ63 ura3Δ0met15Δ0 can1::MFA1pr-HIS3 hht1-hhf1::NatMX4 hht2-hhf2::[HHTS-HHFS]- ura3Δ* H3H39A | OPEN Bio |
| H3R40A | MATa *his3Δ200 leu2Δ0 lys2Δ0 trp1Δ63 ura3Δ0met15Δ0 can1::MFA1pr-HIS3 hht1-hhf1::NatMX4 hht2-hhf2::[HHTS-HHFS]- ura3Δ* H3R40A | OPEN Bio |
| YNL533 | MATa *his3Δ200 leu2Δ0 lys2Δ0 trp1Δ63 ura3Δ0met15Δ0 can1::MFA1pr-HIS3 hht1-hhf1::NatMX4 hht2-hhf2::[HHTS-HHFS]- ura3Δ* H3WT *ρ-* | This Study |
| YNL535 | MATa *his3Δ200 leu2Δ0 lys2Δ0 trp1Δ63 ura3Δ0met15Δ0 can1::MFA1pr-HIS3 hht1-hhf1::NatMX4 hht2-hhf2::[HHTS-HHFS]- ura3Δ* H3K37A *ρ-* | **[1]** |
| YNL592 | MATa *his3Δ200 leu2Δ0 lys2Δ0 trp1Δ63 ura3Δ0met15Δ0 can1::MFA1pr-HIS3 hht1-hhf1::NatMX4 hht2-hhf2::[HHTS-HHFS]- ura3Δ* H3K37A *IXR1-EGFP::KanMX* | This Study |
| YNL595 | MATa *his3Δ200 leu2Δ0 lys2Δ0 trp1Δ63 ura3Δ0met15Δ0 can1::MFA1pr-HIS3 hht1-hhf1::NatMX4 hht2-hhf2::[HHTS-HHFS]- ura3Δ* H3WT *NHP6A-EGFP::KanMX* | This Study |
| YNL596 | MATa *his3Δ200 leu2Δ0 lys2Δ0 trp1Δ63 ura3Δ0met15Δ0 can1::MFA1pr-HIS3 hht1-hhf1::NatMX4 hht2-hhf2::[HHTS-HHFS]- ura3Δ* H3K37A *NHP6A-EGFP::KanMX* | This Study |
| YNL710 | MATa *his3Δ200 leu2Δ0 lys2Δ0 trp1Δ63 ura3Δ0met15Δ0 can1::MFA1pr-HIS3 hht1-hhf1::NatMX4 hht2-hhf2::[HHTS-HHFS]- ura3Δ* H3K37R *NHP6A-EGFP::KanMX* | This Study |
| YNL712 | MATa *his3Δ200 leu2Δ0 lys2Δ0 trp1Δ63 ura3Δ0met15Δ0 can1::MFA1pr-HIS3 hht1-hhf1::NatMX4 hht2-hhf2::[HHTS-HHFS]- ura3Δ* H3K37Q *NHP6A-EGFP::KanMX* | This Study |
| YNL720 | MATa *his3Δ200 leu2Δ0 lys2Δ0 trp1Δ63 ura3Δ0met15Δ0 can1::MFA1pr-HIS3 hht1-hhf1::NatMX4 hht2-hhf2::[HHTS-HHFS]- ura3Δ* H3WT *SPT16-EGFP::KanMX* | This Study |
| YNL721 | MATa *his3Δ200 leu2Δ0 lys2Δ0 trp1Δ63 ura3Δ0met15Δ0 can1::MFA1pr-HIS3 hht1-hhf1::NatMX4 hht2-hhf2::[HHTS-HHFS]- ura3Δ* H3WT *SPT16-EGFP::KanMX* | This Study |
| YNL512 | MATa *his3Δ200 leu2Δ0 lys2Δ0 trp1Δ63 ura3Δ0met15Δ0 can1::MFA1pr-HIS3 hht1-hhf1::NatMX4 hht2-hhf2::[HHTS-HHFS]- ura3Δ* H3WT *nhp6aΔ::KanMX* | This Study |
| YNL514 | MATa *his3Δ200 leu2Δ0 lys2Δ0 trp1Δ63 ura3Δ0met15Δ0 can1::MFA1pr-HIS3 hht1-hhf1::NatMX4 hht2-hhf2::[HHTS-HHFS]- ura3Δ* H3K37A *nhp6aΔ::KanMX* | This Study |
| YNL490 | MATa *his3Δ200 leu2Δ0 lys2Δ0 trp1Δ63 ura3Δ0met15Δ0 can1::MFA1pr-HIS3 hht1-hhf1::NatMX4 hht2-hhf2::[HHTS-HHFS]- ura3Δ* H3WT *nhp10aΔ::KanMX* | This Study |
| YNL491 | MATa *his3Δ200 leu2Δ0 lys2Δ0 trp1Δ63 ura3Δ0met15Δ0 can1::MFA1pr-HIS3 hht1-hhf1::NatMX4 hht2-hhf2::[HHTS-HHFS]- ura3Δ* H3K37A *nhp10aΔ::KanMX* | This Study |
| YNL465 | MATa *his3Δ200 leu2Δ0 lys2Δ0 trp1Δ63 ura3Δ0met15Δ0 can1::MFA1pr-HIS3 hht1-hhf1::NatMX4 hht2-hhf2::[HHTS-HHFS]- ura3Δ* H3WT *hmo1Δ::KanMX* | This Study |
| YNL467 | MATa *his3Δ200 leu2Δ0 lys2Δ0 trp1Δ63 ura3Δ0met15Δ0 can1::MFA1pr-HIS3 hht1-hhf1::NatMX4 hht2-hhf2::[HHTS-HHFS]- ura3Δ* H3K37A *hmo1Δ::KanMX* | This Study |
| YNL469 | MATa *his3Δ200 leu2Δ0 lys2Δ0 trp1Δ63 ura3Δ0met15Δ0 can1::MFA1pr-HIS3 hht1-hhf1::NatMX4 hht2-hhf2::[HHTS-HHFS]- ura3Δ* H3WT *ixr1Δ::KanMX* | This Study |
| YNL471 | MATa *his3Δ200 leu2Δ0 lys2Δ0 trp1Δ63 ura3Δ0met15Δ0 can1::MFA1pr-HIS3 hht1-hhf1::NatMX4 hht2-hhf2::[HHTS-HHFS]- ura3Δ* H3K37A *ixr1Δ::KanMX* | This Study |
| YNL520 | MATa *his3Δ200 leu2Δ0 lys2Δ0 trp1Δ63 ura3Δ0met15Δ0 can1::MFA1pr-HIS3 hht1-hhf1::NatMX4 hht2-hhf2::[HHTS-HHFS]- ura3Δ* H3WT *rox1Δ::KanMX* | This Study |
| YNL522 | MATa *his3Δ200 leu2Δ0 lys2Δ0 trp1Δ63 ura3Δ0met15Δ0 can1::MFA1pr-HIS3 hht1-hhf1::NatMX4 hht2-hhf2::[HHTS-HHFS]- ura3Δ* H3K37A *rox1Δ::KanMX* | This Study |
| YNL537 | MATa *his3Δ200 leu2Δ0 lys2Δ0 trp1Δ63 ura3Δ0met15Δ0 can1::MFA1pr-HIS3 hht1-hhf1::NatMX4 hht2-hhf2::[HHTS-HHFS]- ura3Δ* H3WT *ixr1Δ::KanMX ρ-* | This Study |
| YNL539 | MATa *his3Δ200 leu2Δ0 lys2Δ0 trp1Δ63 ura3Δ0met15Δ0 can1::MFA1pr-HIS3 hht1-hhf1::NatMX4 hht2-hhf2::[HHTS-HHFS]- ura3Δ* H3K37A *ixr1Δ::KanMX ρ-* | This Study |
| YNL547 | MATa *his3Δ200 leu2Δ0 lys2Δ0 trp1Δ63 ura3Δ0met15Δ0 can1::MFA1pr-HIS3 hht1-hhf1::NatMX4 hht2-hhf2::[HHTS-HHFS]- ura3Δ* H3WT *yca1Δ::KanMX* | This Study |
| YNL549 | MATa *his3Δ200 leu2Δ0 lys2Δ0 trp1Δ63 ura3Δ0met15Δ0 can1::MFA1pr-HIS3 hht1-hhf1::NatMX4 hht2-hhf2::[HHTS-HHFS]- ura3Δ* H3K37A *yca1Δ::KanMX* | This Study |
| YNL543 | MATa *his3Δ200 leu2Δ0 lys2Δ0 trp1Δ63 ura3Δ0met15Δ0 can1::MFA1pr-HIS3 hht1-hhf1::NatMX4 hht2-hhf2::[HHTS-HHFS]- ura3Δ* H3WT *nuc1Δ::KanMX* | This Study |
| YNL545 | MATa *his3Δ200 leu2Δ0 lys2Δ0 trp1Δ63 ura3Δ0met15Δ0 can1::MFA1pr-HIS3 hht1-hhf1::NatMX4 hht2-hhf2::[HHTS-HHFS]- ura3Δ* H3K37A *nuc1Δ::KanMX* | This Study |
| YNL575 | MATa *his3Δ200 leu2Δ0 lys2Δ0 trp1Δ63 ura3Δ0met15Δ0 can1::MFA1pr-HIS3 hht1-hhf1::NatMX4 hht2-hhf2::[HHTS-HHFS]- ura3Δ* H3WT *nuc1Δ::KanMX yca1Δ::HphNT1* | This Study |
| YNL576 | MATa *his3Δ200 leu2Δ0 lys2Δ0 trp1Δ63 ura3Δ0met15Δ0 can1::MFA1pr-HIS3 hht1-hhf1::NatMX4 hht2-hhf2::[HHTS-HHFS]- ura3Δ* H3K37A *nuc1Δ::KanMX yca1Δ::HphNT1* | This Study |
| YNL587 | MATa *his3Δ200 leu2Δ0 lys2Δ0 trp1Δ63 ura3Δ0met15Δ0 can1::MFA1pr-HIS3 hht1-hhf1::NatMX4 hht2-hhf2::[HHTS-HHFS]- ura3Δ* H3WT *aif1Δ::KanMX* | This Study |
| YNL589 | MATa *his3Δ200 leu2Δ0 lys2Δ0 trp1Δ63 ura3Δ0met15Δ0 can1::MFA1pr-HIS3 hht1-hhf1::NatMX4 hht2-hhf2::[HHTS-HHFS]- ura3Δ* H3K37A *aif1Δ::KanMX* | This Study |
| YNL726 | MATa *his3Δ200 leu2Δ0 lys2Δ0 trp1Δ63 ura3Δ0met15Δ0 can1::MFA1pr-HIS3 hht1-hhf1::NatMX4 hht2-hhf2::[HHTS-HHFS]- ura3Δ* H3WT *aif1Δ::KanmX nuc1Δ::HphNT1* | This Study |
| YNL727 | MATa *his3Δ200 leu2Δ0 lys2Δ0 trp1Δ63 ura3Δ0met15Δ0 can1::MFA1pr-HIS3 hht1-hhf1::NatMX4 hht2-hhf2::[HHTS-HHFS]- ura3Δ* H3K37A *aif1Δ::KanmX nuc1Δ::HphNT1* | This Study |
| YNL722 | MATa *his3Δ200 leu2Δ0 lys2Δ0 trp1Δ63 ura3Δ0met15Δ0 can1::MFA1pr-HIS3 hht1-hhf1::NatMX4 hht2-hhf2::[HHTS-HHFS]- ura3Δ* H3WT *NHP6A-yeGFP::* *HphNT1* | This Study |
| YNL723 | MATa *his3Δ200 leu2Δ0 lys2Δ0 trp1Δ63 ura3Δ0met15Δ0 can1::MFA1pr-HIS3 hht1-hhf1::NatMX4 hht2-hhf2::[HHTS-HHFS]- ura3Δ* H3K37A *NHP6A-yeGFP:: HphNT1* | This Study |
| YNL724 | MATa *his3Δ200 leu2Δ0 lys2Δ0 trp1Δ63 ura3Δ0met15Δ0 can1::MFA1pr-HIS3 hht1-hhf1::NatMX4 hht2-hhf2::[HHTS-HHFS]- ura3Δ* H3WT *hst4Δ::KanMX NHP6A-yeGFP:: HphNT1* | This Study |
| YNL725 | MATa *his3Δ200 leu2Δ0 lys2Δ0 trp1Δ63 ura3Δ0met15Δ0 can1::MFA1pr-HIS3 hht1-hhf1::NatMX4 hht2-hhf2::[HHTS-HHFS]- ura3Δ* H3K37A *hst4Δ::KanMX NHP6A-yeGFP:: HphNT1* | This Study |
| YNL767 | MATa *his3Δ200 leu2Δ0 lys2Δ0 trp1Δ63 ura3Δ0met15Δ0 can1::MFA1pr-HIS3 hht1-hhf1::NatMX4 hht2-hhf2::[HHTS-HHFS]-HHT1Δ1-32 ura3Δ* | This Study |

**Supplemental Table S2. Yeast plasmids.**

| **Parent Plasmid** | **Vector Description** | **Source** |
| --- | --- | --- |
| pRS416 | *CEN6; ARS4; URA3* | [2] |
| pJU855 | pRS416; SCH9-6xHA (T723D, S726D, T737E, S758E, S765E) | [3] |
| pGAL1-HMO1 | *GAL1prom::HMO1-HA* | Open Biosystems |
| pGAL1-NHP6A | *GAL1prom::NHP6A-HA* | Open Biosystems |
| pGAL1-SOD1 | *GAL1prom::SOD1-HA* | Open Biosystems |
| pGAL1-TIR1 | *GAL1prom::TIR1-HA* | Open Biosystems |
|  |  |  |
| pGAL1-MET10 | *GAL1prom::MET10-HA* | Open Biosystems |
| pGAL1-STF2 | *GAL1prom::STF2-HA* | Open Biosystems |
| pGAL1-PAI3 | *GAL1prom::PAI3-HA* | Open Biosystems |
| pGAL1-YPG1 | *GAL1prom::YPG1-HA* | Open Biosystems |
| pGAL1-PBI2 | *GAL1prom::PBI2-HA* | Open Biosystems |
| pGAL1-YAP1 | *GAL1prom::YAP1-HA* | Open Biosystems |
| pGAL1-TIR3 | *GAL1prom::TIR3-HA* | Open Biosystems |
| pGAL1-GNA1 | *GAL1prom::GNA1-HA* | Open Biosystems |
| pRS425 | *2μ; LEU2* | [2] |
| pSOD1 | *2μ; LEU2; SOD1prom::SOD1* | This study |
| pYDF80 | pRS315*; TOR1-1 (S1972I)* | [4] |

**Supplemental References**

1. Chen, H., et al., Target of rapamycin signaling regulates high mobility group protein association to chromatin, which functions to suppress necrotic cell death. Epigenetics Chromatin, 2013. **6**(1): p. 29.

2. Brachmann, C.B., et al., Designer deletion strains derived from Saccharomyces cerevisiae S288C: a useful set of strains and plasmids for PCR-mediated gene disruption and other applications. Yeast, 1998. **14**(2): p. 115-32.

3. Urban, J., et al., Sch9 is a major target of TORC1 in Saccharomyces cerevisiae. Mol Cell, 2007. **26**(5): p. 663-74.

4. Chan, T.F., et al., A chemical genomics approach toward understanding the global functions of the target of rapamycin protein (TOR). Proc Natl Acad Sci U S A, 2000. **97**(24): p. 13227-32.
